# Asymmetry in three-site relaxation exchange NMR

**DOI:** 10.5194/mr-4-217-2023

**Published:** 2023-08-24

**Authors:** Bernhard Blümich, Matthew Parziale, Matthew Augustine

**Affiliations:** 1 Institut für Technische und Makromolekulare Chemie, RWTH Aachen University, Worringer Weg 2, 52074 Aachen, Germany; 2 Department of Chemistry, UC Davis, One Shields Avenue, Davis, CA 95616, USA

## Abstract

The asymmetry of peak integrals in 2D relaxation maps of exchange between three sites indicates circular flow between the relaxation sites. This disagrees with the detailed balance according to which the exchange between any pair of sites must be balanced in terms of thermodynamic equilibrium. Confined diffusion of particles jumping randomly on a 2D checkerboard grid to any of their eight neighbor positions and confined gas diffusion were modeled in Monte Carlo simulations to explore the impact of topological constraints on particle exchange between three pools. Both models produce density variations across the pore and reveal that up to 1 % of the molecules move in circular paths between the relaxation pools. This motion is driven by different features of either algorithm. It is silent in terms of thermodynamic equilibrium, confirming that multi-site exchange maps are symmetric in this case. The coherent flux is argued to result from stochastic pore resonance related to diffusion eigenmodes. If it can be driven experimentally by external time-varying electric, magnetic, or ultrasonic fields, this may be a way to enhance heterogeneous catalysis.

## 1 Introduction

Exchange is an essential ingredient of diffusion and spreading phenomena, which are abundant in nature and govern the evolution of tangible and intangible objects and goods (Bunde et al., 2018), as well as the physics of living systems (Gnesotto et al., 2018; Lynn et al., 2021). Nuclear magnetic resonance (NMR) provides particularly powerful methodologies to investigate molecular exchange processes (Ernst et al., 1987; Callaghan, 2011). Slow molecular exchange on the timescale of milliseconds is studied by e.g., two-dimensional exchange NMR, i.e., by chemical exchange spectroscopy for rotational motion (Jeener et al., 1979) and by exchange relaxometry for translational motion (Lee et al., 1993). In equilibrium, the nature of the exchange processes is commonly understood to be random Brownian motion, and the associated 2D NMR exchange maps are expected to be symmetric with respect to their diagonal. On the other hand, exchange in non-equilibrium leads to asymmetry. This has been observed in NMR, for example, in 2D chemical exchange spectra for chemical reactions involving different sites (Lacabanne et al., 2022), for the spread of hyperpolarization by spin diffusion (Björgvinsdóttir et al., 2021), for slow flow across porous media in relaxation exchange maps (Olaru et al., 2012), and in position and velocity exchange NMR (Han and Blümich, 2000).

The kinetics of transitions or exchange between discrete states driven by random processes are described by van Kampen (1992) as follows:

1 $$\frac{dM_{i}\left(t\right)}{dt}=\sum_{j}\left\{k_{ij}M_{j}\left(t\right)-k_{ji}M_{i}\left(t\right)\right\},$$

where $M_{i}$ refers to populations represented in NMR by magnetization components collected in the vector M , and $k_{ij}$ refers to the exchange rates equivalent to the transition probabilities from state j to state i , which are collected in the kinetic exchange matrix k . In equilibrium,

2 $$\frac{dM_{i}\left(t\right)}{dt}=0,$$

and the number of all particles arriving at site i from sites j is equal to the number of all particles leaving from site i to sites j so that the total mass is conserved.

As a result of mass balance, two-site exchange between states or sites A and B always leads to symmetric 2D NMR exchange maps in thermodynamic equilibrium as the number $k_{\mathrm{BA}}M_{A}$ of particles populating site B by leaving site A per unit of time is equal to the number of particles $k_{\mathrm{AB}}M_{B}$ leaving site B and populating site A per unit of time. This number is the product of the rate $k_{\mathrm{BA}}$ for transitions from site A to site B times the population $M_{A}$ of site A. The relationship $k_{\mathrm{BA}}M_{A}=k_{\mathrm{AB}}M_{B}$ is known as the principle of detailed balance. In thermal equilibrium, it is understood to also apply to rate processes involving more than two sites (Onsager, 1931; Gnesotto et al., 2018).

As an example of mass-balanced equilibrium diffusion between three sites (Onsager, 1931; Sandstrom, 1983), Eq. (2) becomes

3 $$\begin{array}{rl} & k_{21}M_{1}+k_{31}M_{1}=k_{12}M_{2}+k_{13}M_{3}\\ & k_{12}M_{2}+k_{32}M_{2}=k_{21}M_{1}+k_{23}M_{3}\\ & k_{13}M_{3}+k_{23}M_{3}=k_{31}M_{1}+k_{32}M_{2}, \end{array}$$

or equivalently, mass balance requires

4 $$k_{31}M_{1}-k_{13}M_{3}=k_{12}M_{2}-k_{21}M_{1}=k_{23}M_{3}-k_{32}M_{2}.$$

Normalization of this expression to the total number of exchanges per unit of time defines the asymmetry parameter $a_{\mathrm{sy}}$ used below:

5 $$\left(k_{23}M_{3}-k_{32}M_{2}\right)/\left[\left(1,1,1\right)k\;M\right]\;\;a_{\mathrm{sy}}.$$

Here, $k_{ij}M_{j}$ is the number of transitions from pool j to pool i , corresponding to the peak integral in an exchange map after correction for relaxation effects so that the denominator corresponds to the integral over all peaks. The asymmetry parameter thus quantifies the imbalance of exchange between two sites in terms of the number of unbalanced exchanges normalized to the total number of exchanges. Therefore, it specifies the relative flux in the circular exchange process. While mass balance (Eq. 4) is a necessary condition for dynamic equilibrium, detailed balance, on the other hand, is a stronger condition applicable to thermodynamic equilibrium. It requires the following:

6 $$a_{\mathrm{sy}}=0.$$

Detailed balance was introduced by Maxwell in 1867 based on sufficient reason in his derivation of the speed distribution of gas atoms considering the speed exchange between colliding gas atoms in thermodynamic equilibrium (Maxwell, 1867). An intriguing consequence of the exchange being balanced in detail between particles A and B amounts to the impossibility of assigning positive time to either velocity exchange from A to B or from B to A on the particle scale of the exchange process, thus admitting negative time or time reversal. In 1872, Boltzmann showed, in an elaborate treatment, that Maxwell's speed distribution also applies to polyatomic gas molecules (Boltzmann, 1872). Furthermore, in 1917, Einstein derived Planck's law of black-body radiation as a balanced energy exchange between quantized radiation and matter, underlining the striking similarity to Maxwell's speed distribution of gas atoms (Einstein, 1917). He concludes “Indem Energie und Impuls aufs engste miteinander verknüpft sind, kann deshalb eine Theorie erst dann als berechtigt angesehen werden, wenn gezeigt ist, daß die nach ihr von der Strahlung auf die Materie übertragenen Impulse zu solchen Bewegungen führen, wie sie die Wärmetheorie verlangt” [Since energy and momentum are intimately connected, a theory can only then be considered justified when it has been shown that according to it the momenta of the radiation transferred to the matter lead to such motions as demanded by the theory of heat].

In his work extending Maxwell's speed distribution to polyatomic gas molecules, Boltzmann considered molecules in a container whereby the walls reflected the molecules like elastic balls: “Bezüglich der Gefäßwände, welche das Gas umschließen, will ich jedoch voraussetzen, dass die Moleküle an denselben wie elastische Kugeln reflektiert werden. …Die Wände stören nicht, da an ihnen die Moleküle wie elastische Kugeln reflektiert werden; also geradeso von ihnen zurücktreten, als ob der Raum jenseits der Wände von gleich beschaffenem Gase erfüllt wäre” [Concerning the container walls which enclose the gas, I want to presume that the molecules are reflected from them like elastic balls. …The walls do not interfere because the molecules are reflected from them like elastic balls; that is, they recede from them just like that, as if the space beyond the walls would be filled with similarly conditioned gas]. Moreover, the interaction between gas molecules can be of any type. While Boltzmann states that any other interaction between walls and molecules leads to the same result, albeit at the loss of simplicity, the perfectly elastic reflections of the gas molecules at the walls eliminate the topological constraints of the box on their motion. For confined particles, this means that the pressure across the pore volume is constant; i.e., the time average of the particle density does not vary with the location inside the pore. Boltzmann obtained the same speed distribution for polyatomic molecules with internal degrees of freedom as Maxwell did for atoms based on a detailed balance of speed exchange. In the simulations reported below, the motion of molecules is considered whereby the interactions with the walls are the same as those among the molecules. Understanding confined diffusion (Valiullin, 2017) is important from a general point of view because the motion of molecules without topological constraints is an ideal limit which cannot be perfectly realized in practice, although it may be realized within experimental uncertainty.

**Figure 1:**
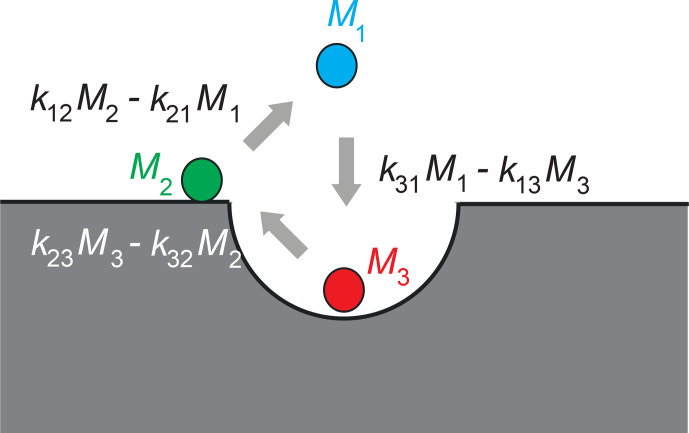
Asymmetry in three-site diffusion-mediated exchange indicates coherent circular motion in a model example of water molecules in contact with a porous surface. Three water populations $M_{j}$ are identified by different NMR relaxation times and colors. They are molecules in the bulk (1), molecules on the surface (2), and molecules in the pores (3). The exchange rate constants are $k_{ji}$ . The net particle flux $k_{ij}M_{j}-k_{ji}M_{i}$ between two sites differs from zero. The net mass of all molecules participating in the exchange is conserved. The figure illustrates positive $a_{\mathrm{sy}}$ .

Two-site exchange processes will always be symmetric in equilibrium. This situation has been evaluated analytically for NMR relaxation exchange of fluids in porous media (McDonald, 2005). Yet, multi-site relaxation exchange NMR maps (Van Landeghem, 2010) can formally be asymmetric in equilibrium. For example, the transverse magnetization $s\left(t_{1},t_{2}\right)$ from a three-site $T_{2}-T_{2}$ relaxation exchange NMR experiment (Gao and Blümich, 2020),

7 $$s\left(t_{1},t_{2}\right)=\left(1,1,1\right)e^{-\left(R_{2}+k\right)t_{2}}e^{-\left(R_{1}+k\right)t_{m}}e^{-\left(R_{2}+k\right)t_{1}}M\left(t_{0}\right),$$

has been simulated to model an experimentally observed asymmetric three-site $T_{2}-T_{2}$ NMR exchange map of water molecules saturating Al ${}_{2}$ O ${}_{3}$ powder, with the three relaxation sites corresponding to bulk water, water molecules on the surface of the powder particles, and water molecules inside the surface pores (Fig. 1). Here, $M\left(t_{0}\right)$ is the initial vector of transverse-magnetization components from relaxation sites 1, 2, and 3 generated from longitudinal thermodynamic equilibrium magnetization with a 90 ${}^{\circ }$ pulse at the beginning of the experiment at time $t_{0}$ , and $t_{1}$ , $t_{m}$ , and $t_{2}$ are the evolution, mixing, and detection time intervals of the 2D NMR experiment, respectively (Callaghan, 2011; Lee et al., 1993). Apart from the relaxation rate matrices $R_{1}$ and $R_{2}$ and the kinetic matrix k , the best match obtained by forward simulation returned the peak integrals, revealing an asymmetry parameter of $a_{\mathrm{sy}}=-1.2$ %. This asymmetry of the forward and backward particle jumps between two sites specifies the relative circular flux between the three sites (Fig. 1).

The asymmetry observed in the experiment can be argued to result from the uncertainty of the measurement and the data processing by 2D inverse Laplace transformation (Song, 2002). Also, asymmetric three-site exchange disagrees with the detailed balance of the exchange between any pair of sites in thermodynamic equilibrium because it needs to be explained by circular diffusion on the pore scale, and such motion resembles that of a rachet, which Feynman has argued disagrees with the second law of thermodynamics (Feynman et al., 1966). Nevertheless, Monte Carlo simulations were executed and are discussed below to investigate asymmetry in three-site exchange.

## 2 Modeling confined diffusion

### 2.1 Vacancy diffusion: random particle jumps on a 2D checkerboard

**Figure 2:**
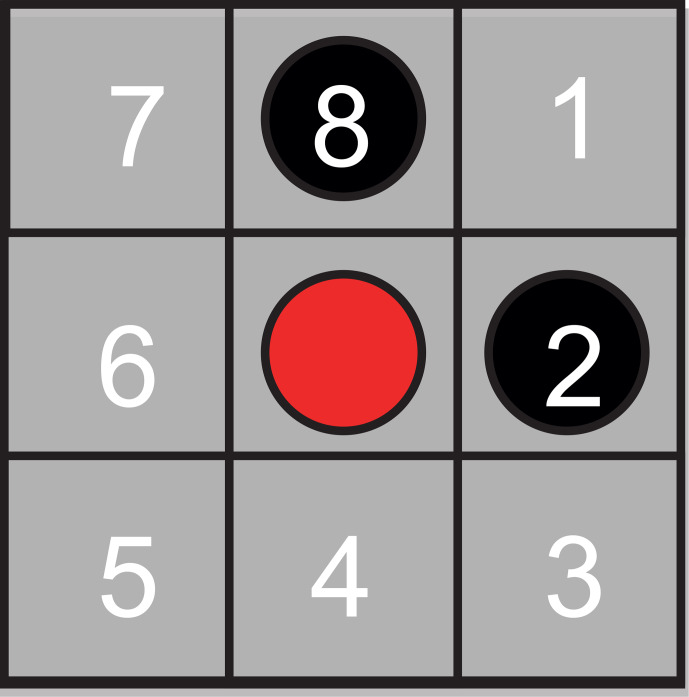
Jumps on a checkerboard grid modeling vacancy diffusion. The center particle can jump to any of its eight nearest neighbor cells, which are numbered clockwise from 1 to 8. Jump probabilities were introduced to account for particle interaction between the center particle (red) and neighboring particles (black).

Random jumps of particles from occupied sites to vacant sites were simulated with a Monte Carlo algorithm (Metropolis et al., 1953; Grebenkov, 2011; Hughes, 1995; Sabelfeld, 1991) in a confined space on a checkerboard. The algorithm models vacancy diffusion (Seitz, 1948) encountered in metals and alloys, but the particles perform the jumps rather than the vacancies. To keep the simulation simple, it is limited to jumps on a 2D 3×3 Moore lattice of range 1 (Wolf-Gladrow, 2000) following the rules of the game of life (Wolf-Gladrow, 2000; Bialynicki-Birula, 2004). Here, the center particle can jump to any of its eight neighbors (Fig. 2). Different neighborhoods of range 1 were tested (Fig. S1 in the Supplement) (Bialynicki-Birula, 2004), but only the Moore neighborhood, having the highest symmetry of all neighborhoods, produced data consistent with Eq. (4). Topological constraints were introduced, setting boundaries for the jump space. Initially, the available cells inside the jump space on the grid were populated randomly with particles up to a specified particle density. Particles in the bulk are indexed as 1, and two distinct boundary sections are indexed as 2 and 3, giving three environments for the particles to be exposed to and between which randomly selected particles can move. A particle jumping from environment j to i is counted by incrementing the element ij of a 3×3 jump matrix with elements $k_{ij}M_{j}$ by 1. If the particle environment does not change with the jump, the respective diagonal element $k_{jj}M_{j}$ is incremented. The NMR relaxation environments are indexed according to increasing relaxation rate. If a particle is in contact with two different relaxation environments, it is assigned to the relaxation environment with the higher index according to the higher relaxation rate.

Different rules governing jumps to a neighbor cell were explored. (1) In the simplest case, one of the eight destination cells was chosen at random without assigning a jump probability. When the destination cell was free, the jump was executed, and the initial and final environments were compared to increment the corresponding entry in the jump matrix accordingly. When the destination cell was occupied, the particle remained at its source cell, and the respective diagonal element of the jump matrix was incremented. In all other cases, jump probabilities were assigned. (2) As a subtle variant of the random jumps to any of the eight neighbor cells, jumps to any of the *free* neighbor cells were randomly selected by assigning zero jump probability to occupied neighbor cells and equal probability for jumps to the empty cells. This algorithm is known to violate detailed balance (Metropolis et al., 1953; Blümich et al., 2023). (3) With reference to the Helmholtz free energy A=U-TS , where U is internal energy, T is temperature, and S is entropy, a jump probability $p=\exp\left\{-\frac{\Delta A}{k_{B}T}\right\}$ was introduced, where ΔA=ΔU-TΔS , T is the temperature, and $k_{B}$ is the Boltzmann constant. ΔU=-FΔR and ΔS were estimated from the sum of distances to free or occupied neighbor cells by crude empirical models, as detailed in the supporting information. Here, F is the force, and ΔR is the distance vector between two particles. This allowed us to probe attractive and repulsive interactions by changing the sign of ΔU in simulation runs and by varying the temperature in addition to varying population density equivalent to pressure. It is noted here that the force field on a randomly populated lattice is not conservative (Blümich et al., 2023). In other words, the energy balance of a particle moving in a circle is different from zero, and Monte Carlo simulations under these constraints probe a driven equilibrium and not the thermodynamic equilibrium (Michel et al., 2014).

The vacancy diffusion simulations were carried out with a program written in MATLAB R2020a by MathWorks on an Apple MacBook Pro 2.4 GHz with an Intel Quad-Core i5 processor. Unless indicated otherwise, 10 ${}^{7}$ jumps were simulated in one run as taking 75 s.

### 2.2 Gas diffusion

The gas diffusion calculations explore similar pore sizes and occupancy. Here, the motion of circular particles with diameters equal to the cell size was accomplished by propagating an initial distribution of particle speeds for random initial positions and directions in a Monte Carlo fashion based on instantaneous collisional forces. This distribution rapidly equilibrated to a Maxwell–Boltzmann distribution. Whereas in vacancy diffusion simulations the distribution of particles in the pore is recorded after each jump, it is recorded in the gas-phase simulations at constant time intervals. If the center of each particle was within one diameter of another, the particles are considered to have collided. Immediately after a collision, the projection of the velocity vector along the collision axis is reversed prior to propagating to the next step. In this approach, the observation time interval must be sufficiently small so that the new velocities are calculated with a small position uncertainty for the colliding particles (Blümich et al., 2023; Michel et al., 2014).

The collisions change both the direction and velocity of the particles at each of the 10 ${}^{9}$ constant time increments used here. Following conservation of momentum and kinetic energy,

8v1,new=v1,old-2m2m1+m2〈v1,old-v2,old,x1-x2〉‖x2-x1‖2x1-x2,9v2,new=v2,old-2m1(m1+m2)〈v2,old-v1,old,x2-x1〉‖x2-x1‖2x2-x1.

These collisions with other particles and the wall are mediated by the particle size, which is set to be a fraction of the pore-side length of one. This means that a square pore with a five-particle diameter side length is populated with particles that have a diameter of one-fifth. To compare the continuous positional output of this model to vacancy diffusion, a two-dimensional square grid with cell size set by the particle diameter is imposed on the entire pore. The quasi-continuous positional output is then binned into these cells and compared to the binned positions from the previous observation to determine if particles translated between the main pore volume, the pore wall, and the active site. The translational information is used to assign estimates of the jump matrix elements and thus the asymmetry parameter $a_{\mathrm{sy}}$ .

The gas diffusion simulations were carried out with a program written in MATLAB R2020a by MathWorks Inc. on a home-built desktop computer possessing an AMD (Advanced Micro Devices) Ryzen 7 2700 processor. In most cases, 10 ${}^{9}$ jumps were simulated in one run, taking roughly 45 h to complete.

## 3 Results

**Figure 3:**
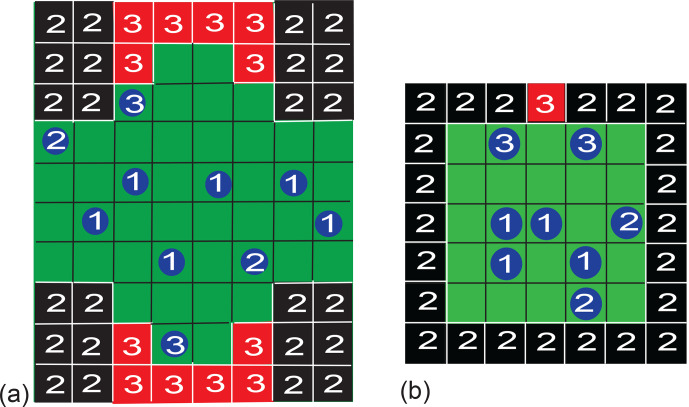
Examples of pore models for two-dimensional three-site exchange based on a checkerboard grid. Particles can occupy one cell and jump to a neighboring one following different realizations of the jump probability. **(a)** Porous solid. The boundaries to the right and left are periodic. The boundaries at the top and bottom are rigid. Depending on their next neighbors in the first coordination shell, the particle relaxation environments are identified as bulk (1), surface (2), and pore (3), with increasing relaxation rates. **(b)** Small square pore with an active site. The bulk (1), the walls (2), and the active site (3) have different relaxation properties. If a particle is in contact with two different relaxation sites, it is counted as belonging to the particle pool with the larger relaxation rate, i.e., the pool with the higher number.

Two different pore geometries were analyzed. Initially, the simulation was executed for a pore geometry (Fig. 3a) which approximated the surface structure of Fig. 1 and which is hypothesized to explain the observed asymmetry of water diffusing in a porous Al ${}_{2}$ O ${}_{3}$ grain pack (Gao and Blümich, 2020). The dented surface was mirrored horizontally to double the probability of particles entering the dent (relaxation site 3) in the otherwise straight surface (relaxation site 2). The bulk of the particles defines relaxation site 1. Periodic boundary conditions were employed to the right and left. A pore boundary was treated just like an occupied cell, with the same rules applying to the jump probability. The simulations of particle motion confined to this complex pore structure and constrained by jump probabilities revealed the existence of asymmetric exchange. To understand the essence of the asymmetry, the pore geometry was simplified to a square with an active site in the wall to study particle motion in detail. Particles in the bulk, in contact with the walls, and in contact with the active site are identified by different NMR relaxation properties (Fig. 3b).

**Figure 4:**
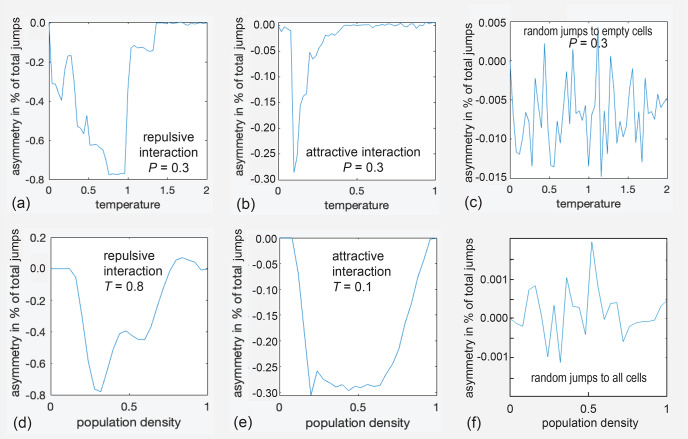
Asymmetry parameters $a_{\mathrm{sy}}$ for diffusion inside the small rectangular pore depicted in Fig. 3b as a function of temperature T **(a–c)** and pressure P **(d–f)**. **(a)** $a_{\mathrm{sy}}(T)$ for repulsive interaction at P=0.3 . **(b)** $a_{\mathrm{sy}}\left(T\right)$ for attractive interaction at P=0.3 . **(c)** $a_{\mathrm{sy}}\left(T\right)$ for jumps to randomly selected empty cells. **(d)** $a_{\mathrm{sy}}\left(P\right)$ for attractive interaction at T=0.8 . **(e)** $a_{\mathrm{sy}}\left(P\right)$ for attractive interaction at T=0.1 . **(f)** $a_{\mathrm{sy}}\left(P\right)$ for jumps to cells randomly selected from all eight neighbor cells.

Enabled by the interaction model, which, depending on the particle environment assigns different jump probabilities as a function of temperature, the asymmetry parameter $a_{\mathrm{sy}}$ was evaluated for both pores with the vacancy diffusion algorithm as a function of temperature T and pressure P . Pressure was varied in terms of the population density, measured as the fraction of cells occupied in the pore. The results for the complex pore are reported in the Supplement (Fig. S3), whereas those for the simple square pore are reported here in the main text (Fig. 4). At certain temperatures and pressures, the autocorrelation function of the occupation-time track of a particular cell and its Fourier transform were also determined. Striking features observed in vacancy diffusion were subsequently modeled with the gas diffusion algorithm in the square pore.

Relevant results for the square pore (Fig. 3b) are summarized in six graphs in Fig. 4. The asymmetry parameter varies strongly with temperature T (Fig. 4a, b) and pressure corresponding to population density P (Fig. 4d, e). All parameters are relative quantities without units. The top three graphs, namely (a), (b), and (c), show the variation of $a_{\mathrm{sy}}$ with temperature for a population fraction of 0.3, corresponding to that of a gas. The asymmetry parameter assumes only negative values in an abrupt but reproducible manner in the range of - 0.8 % < $a_{\mathrm{sy}}$ < 0.0 % for repulsive interaction (Fig. 4a), i.e., for the definition of the force between particles as illustrated in Fig. S2a. With reference to Fig. 1, negative $a_{\mathrm{sy}}$ reports that the straight exit route from the active site towards the center of the pore is preferred over the detour via the pore wall. When the interaction is changed from repulsive to attractive by inverting the sign of ΔU in the expression for the free energy, the asymmetry parameter varies as well, though only between - 0.3 % < $a_{\mathrm{sy}}$ < 0.0 % (Fig. 4b). In either case, the asymmetry parameter varies with temperature and pressure. It is concluded that, for this small pore, up to about 1 % of all jumps on the checkerboard can proceed in an ordered circular fashion between the three sites. Similar behavior is observed for the complex pore of Fig. 3a, as illustrated in Fig. S3 in the Supplement.

At the extrema of the $a_{\mathrm{sy}}\left(T\right)$ curves in Fig. 4a and b, the dependence of the asymmetry parameters on population density was investigated (Fig. 4d, e). The variations with population density are smoother than those with temperature. Significant negative asymmetry results at intermediate pressures, while at low and high pressures, the asymmetry is small (Fig. 4d, e). At higher temperatures and high pressures, small positive $a_{\mathrm{sy}}$ is observed (Fig. 4d, T=0.8 , P=0.8 ). If the destination cell for a jump is chosen at random without considering a hypothetical free-jump energy A , then essentially, noise more than 2 orders of magnitude smaller is observed for the exchange asymmetry determined from 10 ${}^{7}$ jumps when varying T and P (Fig. 4c, f). However, a small bias towards negative $a_{\mathrm{sy}}$ results if the destination cell is chosen at random from all free neighbor cells (Fig. 4c), whereas no bias is detected if the destination cell is chosen at random from all neighbor cells, whether free or occupied (Fig. 4f). This difference becomes more pronounced at a higher number of jumps (see below).

**Figure 5:**
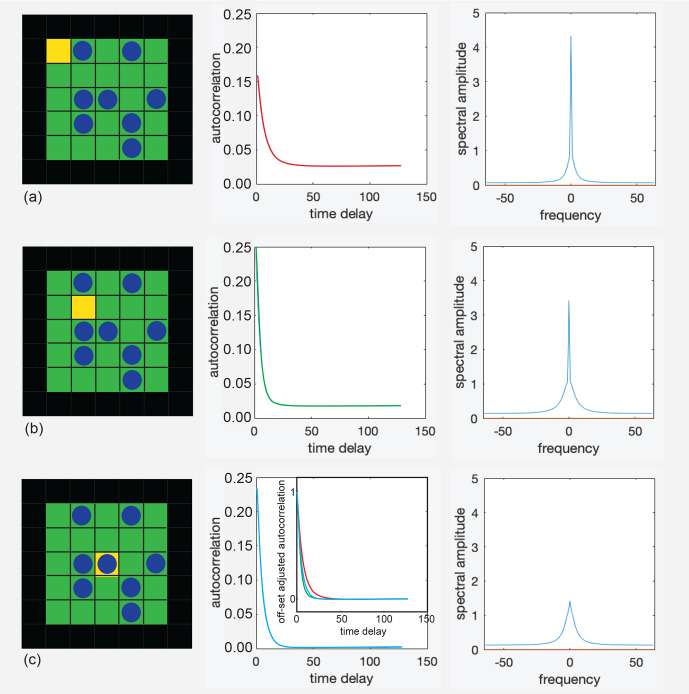
Autocorrelation functions (center) of the occupancy of the yellow cells (left) and the real parts of their Fourier transforms (right) for repulsive interaction at T=0.1 and P=0.3 . **(a)** Corner cell. **(b)** Off-center cell. **(c)** Center cell. The inset in the middle compares the decays of all three autocorrelation functions after subtraction of the offsets.

To shed further light on the origin of the asymmetry, autocorrelation functions of the occupation-time tracks of selected cells in the pore were computed and Fourier transformed (Fig. 5). The occupation-time track was calibrated to zero mean for purely random occupation; i.e., it contained the negative population density when it was empty and the complement of the population density to 1 when the cell was occupied. The faster the autocorrelation function decayed, the less coherently the cell population fluctuated and the broader its Fourier transform was, i.e., the transfer function (Fig. 5b, c). A constant offset of the autocorrelation function shows that the time-average population in the cell differs from the mean population of the pore (Fig. 5a, b). This offset produces a spike at zero frequency in the transfer functions. Subtracting the offsets from the autocorrelation functions and scaling the resulting functions to the same amplitude reveals different decays in different cells and thus variations in particle dynamics across the pore (inset in Fig. 5c, middle). These dynamics cannot readily be measured for a single cell in the pore, although an average over all cells and pores in the measurement volume would be amenable to experimentation by probing the particle dynamics with CPMG (Carr, Purcell, Meiboom, and Gill) measurements in magnetic gradient fields at variable echo times. Such measurements provide the frequency-dependent diffusion coefficient in terms of the Fourier transform of the velocity autocorrelation function (Stepišnik et al., 2014; Callaghan and Stepišnik, 1995; Parsons et al., 2006).

**Figure 6:**
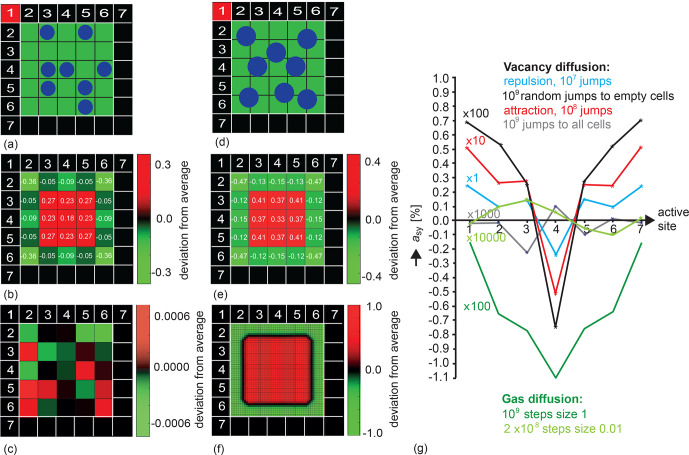
Population density distributions and dependencies of the asymmetry parameter $a_{\mathrm{sy}}$ on the position of the active relaxation site in the wall of a pore with 5×5 cells. **(a)** Vacancy diffusion. Particles can jump one step on the grid in eight directions. **(b)** Deviations from average relative density 1 for 10 ${}^{9}$ jumps chosen at random to any of the free neighbor cells. **(c)** Deviations from average relative density 1 for 10 ${}^{9}$ jumps chosen at random to any of the eight neighbor cells. **(d)** Gas diffusion. The particle motion is computed on a fine grid. **(e)** Deviations from average relative density 1 for 10 ${}^{9}$ observations of particle positions at observation intervals of duration 1. The particle position at the time of observation is binned to the coarse vacancy diffusion grid. **(f)** Deviations from average relative density 1 on a fine 50×50 grid of 0.1 particle diameters for 10 ${}^{9}$ observations of particle positions at observation intervals of duration 0.01. **(g)** Variations of the asymmetry parameter with the position of the active site in the cell wall for differently interacting particles for vacancy diffusion at T=0.2 , P=0.3 , and different jump probabilities, as well as for gas diffusion at long and short observation intervals of 1 vs. 0.01. The mirror symmetry of each trace about the center position reports high precision of the simulation.

While the autocorrelation function is difficult to probe experimentally, the asymmetry parameter $a_{\mathrm{sy}}$ , on the other hand, probes the particle dynamics and could be investigated experimentally directly by relaxation exchange NMR experiments provided the signal-to-noise ratio is good enough. The parameter depends on the location of the relaxation center in the pore wall (Fig. 6). This dependence has been verified to be identical for all walls of the square pore. Moreover, it exhibits mirror symmetry about the center position (Fig. 6g), ensuring that the simulation noise is negligible. For vacancy diffusion in a 5×5 square pore with walls that are seven cells wide (Fig. 6a, b), $a_{\mathrm{sy}}$ varies consistently with position when the jumps are selected following a priori defined probabilities, irrespective of the particle interaction being positive or negative or of the destination cell having been chosen randomly from all free neighbor cells. However, the magnitude of $a_{\mathrm{sy}}$ depends strongly on the selection rule defined by the jump probability, as indicated in Fig. 6g by the scaling factors. It is highest at the corner positions and lowest at the center position. For random jumps to empty cells, $a_{\mathrm{sy}}$ was more than 1 order of magnitude smaller than for repulsive interaction so that the number of particle jumps had to be increased to 10 ${}^{9}$ , resulting in 3 h computation time for each data point in the corresponding trace (Fig. 6e, black). Interestingly, for gas diffusion (Fig. 6d), $a_{\mathrm{sy}}$ varies at long observation intervals (Fig. 6g, green) in a fashion similar to that for vacancy diffusion and is of a magnitude comparable to that of vacancy diffusion (Fig. 6g, black) but does not change sign with the position of the active site in the pore wall. In all these cases, the precision of the asymmetry parameter $a_{\mathrm{sy}}$ obtained in the simulations exceeds the second relevant digit. If the jumps in the vacancy diffusion simulations are chosen without bias from a jump probability, then no exchange asymmetry is detected; only noise nearly more than 1 order of magnitude lower than for jumps selected at random to one of the free neighbor positions is detected (Fig. 6g, gray). Similarly, the asymmetry parameter decreases with the observation time, becoming shorter by more than 2 orders of magnitude, as illustrated in Fig. 6g, for a long time step of 1 (dark green) versus a short time step of 0.01 (light green) in simulation units of (m s ${}^{2}/k_{B}T{)}^{1/2}$ .

The particle dynamics manifested in $a_{\mathrm{sy}}$ are accompanied by variations of the average population density across the pore, which is depleted in the contact layer of the particle with the pore wall, enhanced in the next layer, and tapers off towards the pore center in both cases (Figs. 6b, e, S4). The densities vary in a similar fashion across the pore for both types of diffusion, albeit having somewhat different values, as can be verified by close inspection of the numbers in each cell in Fig. 6b and e. These density variations disagree with Boltzmann's argument that elastic collisions with the walls effectively remove the impact of the walls to the effect that the walls can be neglected. Agreement, however, is reached if the destination cells for particle jumps in vacancy diffusion are chosen at random from all and not just the free neighbor cells (Fig. 6c; Metropolis et al., 1953). Shortening the observation interval in the gas diffusion simulations, however, maintains the unphysical density distribution across the pore and has no effect due to binning of the particle positions to the vacancy diffusion grid at the time of observation as the exact moment of a particle collision cannot be determined on a discrete time axis. On a finer grid, however, the population density is homogeneous, except in the regions close to the walls, which the centers of the circular particles cannot approach (Fig. 6f). If, however, projected onto the coarse vacancy diffusion grid, the population density modulations (Fig. 6e) reappear because the exact locations of collisions cannot be determined in a simple way at finite observation time intervals. Nevertheless, for both algorithms, the asymmetry parameter approaches zero for all positions along the wall of the square pore (Fig. 6g, light green), confirming that detailed balance is observed.

The maps in Fig. 6b, c, e, and f, which reveal the deviation of local population density from average population density, were calculated by summing the 2D maps of particle locations after each jump or at each observation time, normalizing the resultant maps to the number of jumps and the particle density and subtracting the average mean expected for a constant particle density across all cells in the pore. Further maps of population density variations for the two different pores of Fig. 3 with other sizes and interaction parameters are summarized in Fig. S4 of the Supplement. While the particle density varies less with temperature for vacancy diffusion, different density patterns are found at different pressures. The strongest density variations are near the pore wall regardless of whether the interaction is repulsive, attractive, or based on prior knowledge that a neighbor cell is occupied. This becomes particularly evident for larger pores (Fig. S4b, d, e). Coincidentally, at low density, the main features of the density maps are strikingly similar for vacancy diffusion with destination cells chosen randomly from among the free neighbor cells (Fig. S4b) and for gas diffusion (Fig. S4d). The particle density is strongly depleted at the pore corners and near the wall and is significantly increased in the next particle layer (Fig. S4e, f). For interacting particles, this concentration variation is carried forwards in vacancy diffusion with increasing distance from the wall, leading to concentration waves which taper off towards the center of the pore and interfere with each other coming from different directions. For small pores, interference patterns dominate the density distribution across the pore (Figs. 6b, e and S4a, c). For particles jumping randomly to empty neighbor cells, the decay of the concentration wave towards the pore center is fast, with few to no oscillations towards the pore center, while the oscillations are enhanced by conditioning the jump probability with a hypothetical free-jump energy (Fig. S4d, P=0.2 ). In particular, the population density at the active site in the dent of the complex pore of Fig. 3a depends on the parameters P and T (Fig. S4a, b).

## 4 Discussion

Confined two-dimensional diffusion has been modeled by two different algorithms to investigate to what extent the cross-peaks in 2D $T_{2}-T_{2}$ exchange maps can be asymmetric. The asymmetry is quantified by an asymmetry parameter $a_{\mathrm{sy}}$ , which indicates the relative flux between two sites corresponding to the difference in the number of forward and backward exchanges normalized to the total number of exchanges. The vacancy diffusion algorithm models particle jumps on a checkerboard grid to the nearest neighbor cells under the constraint of different jump probabilities and samples the population map after each jump. The jump probability was determined from a Boltzmann distribution with a heuristic free energy which depends on the populations of the surrounding cells. The asymmetry parameters turned out to be equal to zero in the case of equal jump probability to all neighbor cells (Metropolis et al., 1953), whether occupied or not, confirming the validity of detailed balance (Fig. 6g). They were found to be different from zero when different jump probabilities were assigned to different neighbor cells, i.e., when the jump energy depended on the population pattern of the neighbor cells. However, with the statistical arrangement of the particles on a checkerboard and the confinement of the interaction force to the next-nearest neighbors, energy was not conserved with a particle move so that each particle move either injected or extracted energy from the system. Nevertheless, the equilibrium condition (Eqs. 2 and 3) was fulfilled so that the system was not in thermodynamical equilibrium but rather in an equilibrium that was driven by the algorithm. The observed asymmetry parameter was, therefore, assigned to a driven and not thermodynamic equilibrium.

The gas diffusion algorithm models particles colliding with initial velocity vectors and calculates new velocity vectors after a collision from conservation of energy and momentum, whereby the instant of a collision is interrogated on a discrete time grid. The smaller the observation time, the more precisely the instant of a collision is determined. Any deviation from the exact collision time leads to errors in the position coordinates of the colliding particles and thus their velocities (Eqs. 7, 8; Michel et al., 2014). While for large observation times a significant asymmetry parameter is observed (Fig. 6g, dark green), its value shrinks drastically when the observation time is reduced by a factor of 100 (Fig. 6g, light green). It is concluded that, with the limit of infinitely short observation time, the gas diffusion algorithm can also produce a vanishing asymmetry parameter in a three-site exchange in agreement with the principle of detailed balance and with symmetry in the cross-peak intensities of exchange maps in thermodynamic equilibrium. If, on the other hand, the velocities are calculated with a systematic error in the gas diffusion model due to a finite observation interval, the resultant velocities disagree with the energy and momenta of elastic collisions so that, here, energy is also injected or extracted from the system, and the observed asymmetry parameter can be attributed to a driven and not a thermodynamic equilibrium.

The asymmetry parameters observed for either of the two pore shapes (Fig. 3) investigated with the vacancy diffusion model vary in a range on the order of $-1\;\%<a_{\mathrm{sy}}<1\;\%$ ; i.e., up to 1 % of all particles in the pore do not follow the detailed balance between all pairs of sites but move coherently in circles between the three sites. It is emphasized that this circular exchange is between the pools of particles representing the three sites, and it is not a motion followed by individual particles completing circular jumps. Given repulsive or attractive interaction in the vacancy diffusion model with heuristic temperature- and pressure-dependent jump probabilities, the variations of $a_{\mathrm{sy}}$ with temperature T appear to be rapid, reminiscent of phase transitions (Figs. 4a, b, S3a). The variations of $a_{\mathrm{sy}}$ with pressure corresponding to population density P are smooth (Figs. 4d, e, S3b). Either positive or negative values of $a_{\mathrm{sy}}$ are observed as T or P change. A sign change of $a_{\mathrm{sy}}$ indicates a change in the sense of the circular exchange (see Fig. 1).

For a simple square pore, the asymmetry parameter varies with the position of the active site in the cell wall, exhibiting mirror symmetry with respect to the wall center (Fig. 6g). The variation is the same for the different jump probabilities, referred to as repulsive and attractive interaction or random jumps to empty cells; albeit, it differs significantly in magnitude. A similar dependence is observed in the gas-phase diffusion simulations at long observation times. Moreover, the autocorrelation functions and their Fourier transforms have been determined for the occupancy time tracks of selected cells at specific positions inside a small square pore for 10 ${}^{7}$ jumps of all particles in the pore (Fig. 5). The time track function was devised to have zero mean for the average cell population. Depending on the position of the cell inside the pore, the autocorrelation functions and their Fourier transforms vary. Specifically, the autocorrelation function can exhibit a significant constant offset. At these positions inside the pore, the particle densities are different from the pore average, and the cell is, on average, emptier or more occupied than would be expected if the exchange between all cells were the same. This conclusion is supported by the observed deviations of the cell occupancies from the pore average (Figs. 6b, e, S4). Near the pore wall, the average population density is depleted and varies in an oscillatory manner along the pore wall. Further towards the center of the pore, the average population density increases sharply and then tapers off towards the pore center to a value slightly above the average density.

These observations for driven vacancy diffusion in a square pore with 5×5 cells are compared to independent simulations of driven gas diffusion (long observation time – step size 1) of non-interacting particles in a square pore with an edge length of five particle diameters and that also allows for seven relaxation centers along the pore wall (Fig. 6a, d). A similar variation of the asymmetry parameter is found for vacancy diffusion, but the asymmetry parameter is negative for all positions of the active site (Fig. 6g, dark green). Moreover, the depletion of the average particle density at the pore wall and its subsequent variation towards the center are similar, with the exception that oscillations of the average particle density along the pore wall are weaker for gas diffusion (Fig. 6b, e). These oscillations persist even at short observation times due to the uncertainty of localizing the particle positions at the exact time of their collision on a discrete time grid. The lack of a sign change in the asymmetry parameter with changing position of the active site may be explained by the destructive interference of particle collisions from multiple sites with the wall within one discrete particle diameter and the fact that the free path length between collisions in gas diffusion is not limited to the next cell as in vacancy diffusion but can range up to the pore diameter. Taken together, the observed asymmetry in the three-site exchange in driven equilibrium and the variation of the jump statistics with position inside the pore point to diffusive resonance phenomena like standing waves of air in pipes, as reported by Kundt (Kundt, 1866), or vibrating plates, as reported by Chladni (Chladni, 1787).

Three-site exchange can be viewed as a finite-difference approximation to the Laplace operator (van Kampen, 1992; Kuprov, 2022) governing Fick's second law (Fick, 1855). Considering some local site N with neighbor sites N-1 and N+1 to the right and left, the mass flow to and from site N given by Eq. (1) is

10 $$\begin{array}{rl} \frac{dm_{N}\left(t\right)}{dt} & =k_{N,N-1}m_{N-1}-k_{N-1,N}m_{N}\\ & +k_{N,N+1}m_{N+1}-k_{N+1,N}m_{N}. \end{array}$$

Taking the limit to the infinitesimally small distance Δr→dr between the neighboring sites leads to $k_{j,i}=k$ , revealing that Eq. (10) is a finite-difference approximation of a second spatial derivative balanced by the temporal variations of m during infinitesimal time dt :

11 $$\left(k\;m_{N-1}-2\;k\;m_{N}+k\;m_{N+1}\right)/\Delta r^{2}\approx k\frac{d^{2}m}{dr^{2}}=\frac{dm}{dt}/\Delta r^{2}.$$

In this limit, Eq. (11) becomes Fick's second law, with the diffusion coefficient $D=k\Delta r^{2}$ . This back-of-the-envelope argument suggests that the observed asymmetry of the three-site exchange is a property of Fick's second law and relates to eigenmodes of the Laplace operator (de Hoop and Prange, 2007; Grebenkov and Nguyen, 2013).

**Figure 7:**
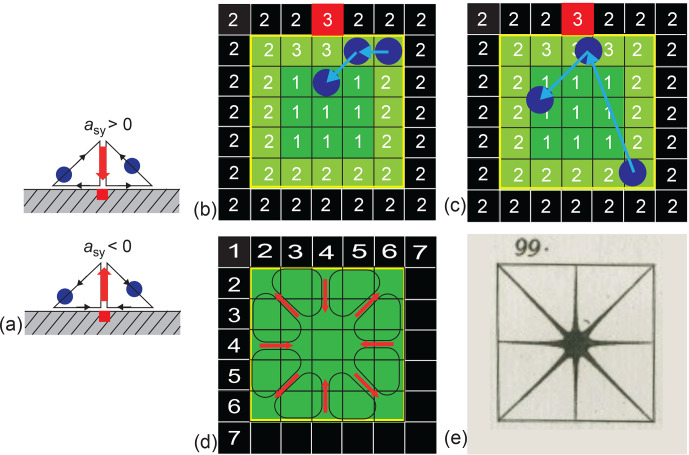
Illustration of the exchange asymmetry in driven equilibrium for the square pore of Fig. 6a. **(a)** Depending on the sign of the asymmetry parameter, a small fraction of diffusing particles (blue circles) prefer the direct path towards or away from the active site (red square) at the pore boundary over the path along the boundary from the active site. **(b)** Vacancy diffusion for negative asymmetry parameter and the active site 3 in the center of the pore wall. Jumps are limited to the next-nearest cells. The direct path away from the active site to the bulk 1 in the center is preferred over the path along the pore wall 2 when leaving the region in contact with the active site. **(c)** Gas diffusion for negative asymmetry parameter and the active site 3 in the center of the pore wall. The free paths between collisions can span the entire cell. **(d)** In-plane translational vacancy diffusion paths resulting from the variation of the asymmetry parameters with the position of the active site on the pore wall depicted in Fig. 6g. **(e)** Out-of-plane vibrational mode of a square plate observed by Chladni (Chladni, 1787).

The diffusion equation applicable to longitudinal magnetization in NMR instead of particle masses m is the Bloch–Torrey equation (Torrey, 1956):

12 $$\frac{\partial }{\partial t}m\left(r,t\right)=D\nabla^{2}m\left(r,t\right)-\mu \;m\left(r,t\right),$$

where m now is the magnetization deviation from thermal equilibrium, and μ is the bulk relaxation rate. $m\left(r,t\right)$ solves this equation in terms of an expansion into normalized eigenfunctions $\phi_{n}\left(r\right)$ with amplitudes $A_{n}$ and eigenvalues $\tau_{n}$ (Brownstein and Tarr, 1977; Song, 2000):

13 $$m\left(r,t\right)=e^{-\mu t}\sum_{n=0}^{\infty }A_{n}\phi_{n}\left(r\right)\;e^{-\frac{t}{\tau_{n}}}.$$

The eigenvalues are determined by the boundary condition

14 $$D\;n\;\nabla \phi_{n}\left(r\right)=\rho \;\phi_{n}\left(r\right),$$

where ρ is the surface relaxivity, and n is the unit vector normal to the surface. They depend on the diffusion coefficient and determine the NMR relaxation time in different ways according to the pore geometry. The population $\phi_{0}$ of the lowest normal mode has no nodes. The higher normal modes $\phi_{n}$ possess nodal surfaces. The higher diffusion eigenmodes have been detected by NMR with selective excitation of partial pore volumes making use of field gradients internal to the pore (Song, 2000). These experimental results reported by Song agree with the Monte Carlo simulations of driven diffusive translational motion in pores reported here in that the population density varies across the pore, and the offset of the autocorrelation function of the local pore occupancy depends on the position of the cell in the pore. It needs to be investigated further how much the NMR relaxation times and the associated particle dynamics vary with the position from the pore wall to the center in the driven concentration wave (Bytchenkoff and Rodts, 2011). On the other hand, stochastic resonance in thermodynamic equilibrium was observed with NMR first by Sleator et al. (1985) and subsequently studied in detail in different scenarios by Müller and Jerschow (2005) and Schlagnitweit and Müller (2012). There, the magnetization fluctuating with the thermal motion of the nuclear spins assumes the role of the particles, and the resonance circuit assumes the role of the pore. Diffusion eigenmodes are expected to be unobservable with this method unless a subset of modes is driven by an external stimulus because they may be silent in thermodynamic equilibrium.

From the exchange asymmetry of the particles in the square pore investigated in Fig. 6, a suggestive picture emerges for driven confined vacancy diffusion (Fig. 7), where the diffusion lengths are confined to the distances from the particle to the direct neighbor cells. Depending on the sign of the asymmetry parameter (Fig. 7a), a small fraction of the particles (blue circles) prefer the direct path towards or away from the active site (red square) at the pore boundary over the path along the boundary to or from the active site. In the center of the wall, the direct path away from the active site to the bulk is preferred over the path along the pore wall when leaving the region in contact with the active site (Fig. 7b). But because jumps are allowed only to neighboring cells in vacancy diffusion, the cells belonging to relaxation pool 2 at the wall to the right and left of the active site 3 must be populated from the bulk 1 by direct jumps from the bulk to the wall. For these jumps, the asymmetry parameter is indeed positive, as observed for the off-center positions of the active site (Fig. 6g). Given the symmetry of the square pore, the in-plane translational diffusion paths resulting from the variation of the asymmetry parameters with the position of the active site on the pore wall demand the existence of eight diffusion vortices inside the planar pore (Fig. 7d). The symmetry of this in-plane translational diffusion pattern matches the symmetry of one of the node patterns of the out-of-plane vibrational modes of a square plate observed by Chladni (Fig. 7e) about a quarter of a millennium earlier (Chladni, 1787). This also suggests that the dynamic of driven vacancy diffusion observed in the computer model reported here is a resonance feature of the pore and thus relates to diffusion eigenmodes. The resonance effect is less pronounced for gas diffusion (Fig. 7c) where the free paths between collisions can span the entire cell. Because the mass flow from relaxation site 2 to the active site 3 can be sustained from any position at the pore wall, the asymmetry parameter does not need to change sign when the active site moves along the pore wall (Fig. 7e), and the circular paths can have various shapes and can extend across the entire pore so that the vortex pattern is largely washed out.

Given the technological importance of fluid motion in small pores in heterogeneous catalysis (Kärger et al., 2012), it will be interesting to explore whether such a correlated motion resulting from standing longitudinal particle concentration wave patterns near pore walls can be driven by external stimuli like ultrasound, electric, or magnetic fields. The standing waves could be enhanced by tuning the driver frequency to the pore resonance like a musician enforces resonance modes on a flute when playing. To enhance the resonance modes, low-power, broadband, forced oscillations can also be considered, such as in Fourier transform infrared spectroscopy (Michelson, 1903) and stochastic NMR spectroscopy (Ernst, 1970), while triggering free oscillations by means of high-power impulses may destroy the porous medium under study.

## 5 Summary

The evidence provided by Monte Carlo simulations of random particle jumps on a 2D checkerboard and by simulations of 2D gas diffusion with topological confinements supports the notion that asymmetry in three-site exchange maps is an indication of the non-Brownian diffusion dynamics of confined particles in driven equilibrium. Depending on the sign of the asymmetry parameter, a small fraction of all particles prefers the direct path towards or away from the active site at the pore boundary over the path along the boundary to or from the active site, resulting in a circular flux (Fig. 7). Both driven vacancy diffusion and driven gas diffusion produce congruent results. These are as follows: (1) circular exchange is a manifestation of driven equilibrium and leads to asymmetry of exchange peaks, while thermodynamic equilibrium manifests itself in the detailed balance and symmetry of exchange peaks. (2) The circular exchange in driven equilibrium appears to be a resonance phenomenon which can potentially be driven by external stimuli. Yet, the reported simulations are limited to two dimensions, and it may be argued that the asymmetry of exchange vanishes in the more common pores with three spatial dimensions. However, two-dimensional diffusion is not an abstract model and arises for gas atoms adsorbed to metal surfaces (Oura et al., 2013) so that the driven coherent particle diffusion indicated by the non-zero asymmetry parameter may be observed there. Given the congruent simulation evidence for driven vacancy diffusion and gas diffusion in two-dimensional confinements, it is hypothesized that confined diffusion can be partially converted to coherent motion by external excitation so that the detailed balance will be violated as observed in nonequilibrium phenomena (Gnesotto et al., 2018; Lynn et al., 2021).

## Supplement

10.5194/mr-4-217-2023-supplement The supplement related to this article is available online at: https://doi.org/10.5194/mr-4-217-2023-supplement.

## Data Availability

The reported data can be reproduced with the software codes supplied in the Supplement and the parameters given in the text.

## Code availability

The codes for simulating confined 2D vacancy diffusion and confined 2D gas diffusion are reported in the Supplement.
