# Supplementary material for: Asymmetry in three-site relaxation exchange NMR
Source: Magn Reson (Gott). 2023 Aug 24;4(2):217–29. doi: 10.5194/mr-4-217-2023 (PMC10539757; doi:10.5194/mr-4-217-2023)
Supplement: The supplement related to this article is available online at: https://doi.org/10.5194/mr-4-217-2023-supplement. [file mr-4-217-supplement.pdf]

Supplement of Magn. Reson., 4, 217–229, 2023
https://doi.org/10.5194/mr-4-217-2023-supplement
© Author(s) 2023. CC BY 4.0 License.

Supplement of

Asymmetry in three-site relaxation exchange NMR

Bernhard Blümich et al.

Correspondence to: Bernhard Blümich (bluemich@itmc.rwth-aachen.de)

The copyright of individual parts of the supplement might differ from the article licence.

Neighborhoods explored in 2D vacancy-diffusion simulations

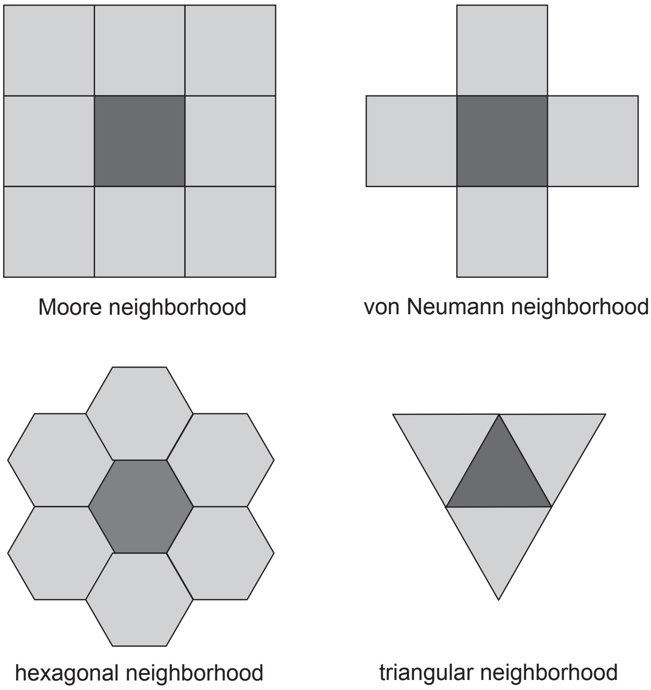

Figure S1. Simulation neighborhoods (grids) of range 1 for jumps from the center position (dark grey) to neighbor positions (light grey). All three asymmetry parameters from Eqn. (4) were calculated at each simulation run. Only the vacancy-diffusion simulations produced with the Moore neighborhood obeyed Eqn. (4), while all gas-diffusion simulations did.

Empirical Ansatz for the estimation of the transition probability from ΔU and ΔS

In an effort to introduce interactions between a particle and its surroundings, a quantity reminiscent of the free energy change $\Delta A = \Delta U - T\,\Delta S$ is determined from crude models of the internal energy change $\Delta U = -\boldsymbol{F}\,\Delta\boldsymbol{R}$ defined by the net force $\boldsymbol{F}$ exerted from all neighboring particles on the particle at stake and the length $|\Delta\boldsymbol{R}|$ of the jump to the next cell, the temperature T, and the entropy change ΔS. The force $\boldsymbol{F}$ between two particles follows Newton's inverse square distance law. It is proportional to $\frac{1}{|\Delta\boldsymbol{R}|^2}$ in the direction of $\frac{1}{|\Delta\boldsymbol{R}|}\Delta\boldsymbol{R}$ from an occupied cell j to the particle i under consideration. The total force the particle i experiences is estimated from the vector sum of the forces exerted from the particles j in all occupied neighbor cells (Fig. 2a),

$$\boldsymbol{F}_i = \sum_j \frac{1}{|\Delta\boldsymbol{R}_{j,i}|^3}\Delta\boldsymbol{R}_{j,i}. \quad (1)$$

The internal energy change $\Delta U_{f,i} = -(\boldsymbol{F}_f - \boldsymbol{F}_i)\Delta\boldsymbol{R}_{f,i} \approx \boldsymbol{F}_i\Delta\boldsymbol{R}_{f,i}$ is modeled for each potential jump from the initial, occupied cell i to the final, empty cell f by the product

of the net force $\boldsymbol{F}_i$ with the vector $\Delta\boldsymbol{R}_{f,i}$ connecting the centers of the initial cell i and the final cell f.

The entropy change $\Delta S = S_f - S_i$ is the difference between the entropies of the particle with its eight nearest neighbors for the final state f and the initial state i. It is modeled by the sum of the step lengths $R_{f,i} = |\Delta\boldsymbol{R}_{f,i}|$ of the particle i to its unoccupied next nearest neighbor cells f,

$$S_i = \sum_f \Delta R_{f,i}. \quad (2)$$

In case a neighbor cell is occupied, $\Delta\boldsymbol{R}_{f,i} = 0$. Detailed examples are worked out below.

The sum of distances has been used as a model for the configurational entropy $S = -k_\mathrm{B}\Sigma_n P_n \ln(P_n)$, because the configurations on the square grid are discrete and differ so that the standard formula $S = k_\mathrm{B}\ln(W)$ does not strictly apply. The sum of jump distances in the Moore neighborhood can be argued to approximate W (but not the logarithm) apart from some scaling factor. This crude approximation exhibits the essential features of entropy: The distance sum is zero, if there is only one possible configuration, and it grows with the number of accessible configurations. For purpose of calculating a jump probability $p = \exp\left\{-\frac{\Delta A}{k_B T}\right\}$ this suffices.

In each jump step, an initially occupied cell i is selected at random and p is evaluated for all possible jumps to neighboring empty cells as potential final cells f. If for one or more jumps $p \geq 1$ is calculated, p is set to 1 and the destination cell of the jump picked at random from this subset of all potential jumps. If all neighbor cells are occupied, $p = 0$, and no jump is counted. If $0 < p < 1$ the destination cell is chosen at random from all those with the same largest jump probability $p < 1$. In the reported simulations, the Boltzmann constant k_B has been set to 1 and so has the shortest distance between neighboring cells.

Two other jump algorithms for choosing the destination cell were also tested: 1) Equal probability for all unoccupied neighbor cells, assigning jump probability zero to occupied cells, and 2) equal probability for choosing the destination cell from all neighbor cells. Respective results are reported in the main body of the manuscript.

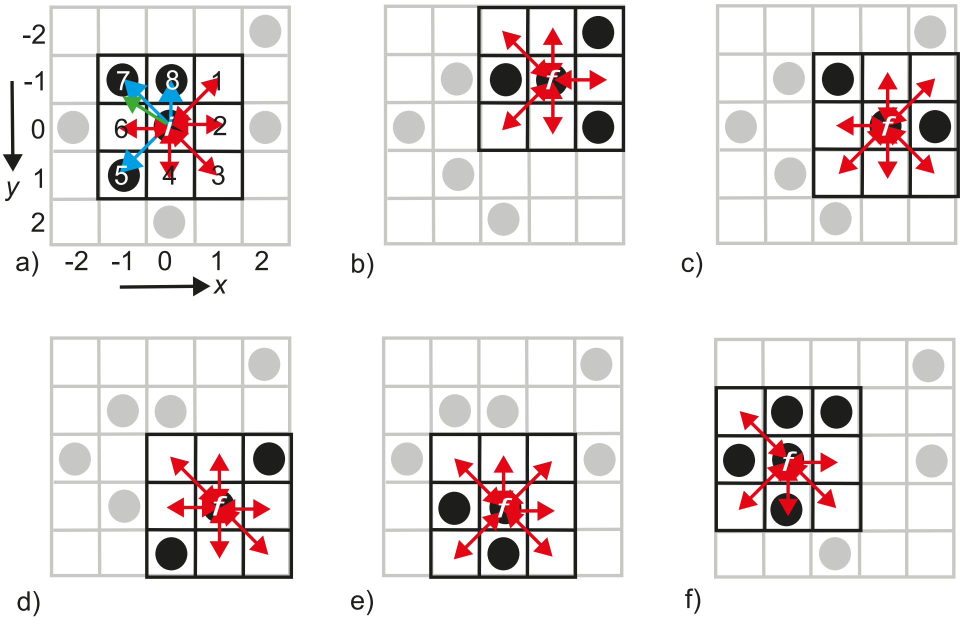

Figure S2. Checkerboard randomly occupied by particles represented by filled circles. a) The cells surrounding the initial particle position i are numbered clockwise from 1 to 8. Cells 5,7, and 8 are occupied, the others are empty. The force (green arrow) on the center particle is calculated as the sum of forces exerted from all particles in the occupied nearest neighbor cells (blue arrows). The entropy is estimated from the sum of distances to all neighboring free cells (red double arrows). b-f) The center particle in a) can jump to any of the free cells 1, 2, 3, 4, and 6, each of which has its own entropy. The final position f of the jump is identified with a bias given by the jump probability based on a simple heuristic model of the free jump-energy difference.

Example (Fig. S2):

- Calculation of internal energy change $\Delta U_{f,i} = -(\boldsymbol{F}_f - \boldsymbol{F}_i)\Delta \boldsymbol{R}_{f,i} \approx \boldsymbol{F}_i \Delta \boldsymbol{R}_{f,i}$:

$$\boldsymbol{F}_i = \left[0+0+0+0+\frac{1}{2^{3/2}}\begin{pmatrix}-1\\1\end{pmatrix}+0+\frac{1}{2^{3/2}}\begin{pmatrix}-1\\-1\end{pmatrix}+\begin{pmatrix}0\\-1\end{pmatrix}\right]=\begin{pmatrix}-2^{-1/2}\\-1\end{pmatrix},$$

$$\Delta\boldsymbol{R}_1=\begin{pmatrix}1\\-1\end{pmatrix},\Delta\boldsymbol{R}_2=\begin{pmatrix}1\\0\end{pmatrix},\Delta\boldsymbol{R}_3=\begin{pmatrix}1\\1\end{pmatrix},\Delta\boldsymbol{R}_4=\begin{pmatrix}0\\1\end{pmatrix},$$

$$\Delta\boldsymbol{R}_5=\begin{pmatrix}0\\0\end{pmatrix},\Delta\boldsymbol{R}_6=\begin{pmatrix}-1\\0\end{pmatrix},\Delta\boldsymbol{R}_7=\begin{pmatrix}0\\0\end{pmatrix},\Delta\boldsymbol{R}_8=\begin{pmatrix}0\\0\end{pmatrix}.$$

The values of ΔU for the 8 neighboring cells are

$$\Delta U = \left\{-2^{-\frac{1}{2}}+1,-2^{-\frac{1}{2}},-2^{-\frac{1}{2}}-1,-1,0,2^{-\frac{1}{2}},0,0\right\}.$$

- Estimation of entropies S from the distances $|\Delta \boldsymbol{R}|$ to all 8 neighbors in the particle-centered tic-tac-toe frame:

Initial state (Fig. 2a): $S_i = 2^{\frac{1}{2}}+1+2^{\frac{1}{2}}+1+0+1+0+0 = 3+2\,2^{\frac{1}{2}}$

Final state 1 (Fig. 2b): $S_f = 0+1+0+1+2^{\frac{1}{2}}+0+2^{\frac{1}{2}}+1 = 3+2\,2^{\frac{1}{2}}$

Final state 2 (Fig. 2c): $S_f = 2^{\frac{1}{2}}+0+2^{\frac{1}{2}}+1+2^{\frac{1}{2}}+1+0+1 = 3+3\,2^{\frac{1}{2}}$

Final state 3 (Fig. 2d): $S_f = 0+1+2^{\frac{1}{2}}+1+0+1+2^{\frac{1}{2}}+1 = 4+2\,2^{\frac{1}{2}}$

Final state 4 (Fig. 2e): $S_f = 2^{\frac{1}{2}} + 1 + 2^{\frac{1}{2}} + 0 + 2^{\frac{1}{2}} + 0 + 2^{\frac{1}{2}} + 1 = 2 + 4\,2^{\frac{1}{2}}$

Final state 5: $S_f = 0$

Final state 6 (Fig. 2f): $S_f = 0 + 1 + 2^{\frac{1}{2}} + 0 + 2^{\frac{1}{2}} + 0 + 2^{\frac{1}{2}} + 0 = 1 + 3\,2^{\frac{1}{2}}$

Final state 7: $S_f = 0$

Final state 8: $S_f = 0$

The possible entropy changes are

$$\Delta S = S_f - S_i = \left\{0, \sqrt{2}, 1, -1 + 2\,2^{\frac{1}{2}}, -3 - 2\,2^{\frac{1}{2}}, -2 + 2^{\frac{1}{2}}, -3 - 2\,2^{\frac{1}{2}}, -3 - 2\,2^{\frac{1}{2}}\right\}.$$

Temperature and pressure dependences of exchange in the complex pore

Relevant results for the pore structure of Fig. 3a are summarized in six graphs in Fig. S3. All parameters are relative quantities without units. The top three graphs a), b) and c) show the variation of a_{sy} with temperature for a population fraction of 0.2 corresponding that of a gas. The asymmetry parameter assumes positive and negative values in a seemingly erratic but reproducible manner in the range of -0.7% < a_{sy} < 0.4% for repulsive interaction (Fig. S3a), i. e. for the definition of the force between particles as illustrated in Fig. 2a. The interaction can be changed to attractive by changing the sign of ΔU in the expression for the free energy. In this case the asymmetry parameter varies as well, however, only between ≈0% < a_{sy} < 0.5% (Fig. S3b). In either case, up to roughly 0.5% of all jumps on the checkerboard proceed in a circular fashion between the three sites. With reference to Fig. 1, positive a_{sy} reports that the straight entry route from the bulk into the small pore is preferred over the detour via the grain surface. This is the case for attractive interaction at $T < 2$ (Fig. S3b). For repulsive interactions and temperatures $T > 1$, a_{sy} is negative and the opposite route is preferred (Fig. S3a). If the destination cell is chosen at random from all free neighbor cells, then the simulation produces largely noise for a_{sy} (Fig. S3c). The noise level is two orders of magnitude smaller than the maximum absolute values of a_{sy} obtained with either repulsive (Fig. S3a) or attractive interaction (Fig. S3b).

At the extrema of the $a_{\mathrm{sy}}(T)$ curves in Figs. S3a,b the dependences of the asymmetry parameters on pressure corresponding to population density were investigated (Figs. S3d-f). The variations with population density are smoother than those with temperature. Positive and negative values of a_{sy} result at a low temperature of $T = 0.2$ for repulsive interaction (Fig. S3d), whereas either negative or positive

values arise for repulsive (Fig. S3e) and attractive (Fig. S3f) interactions at higher temperatures of $T = 2.2$ and 1.3, respectively. Interestingly, two well developed positive modes result for attractive interaction at $T = 1.3$.

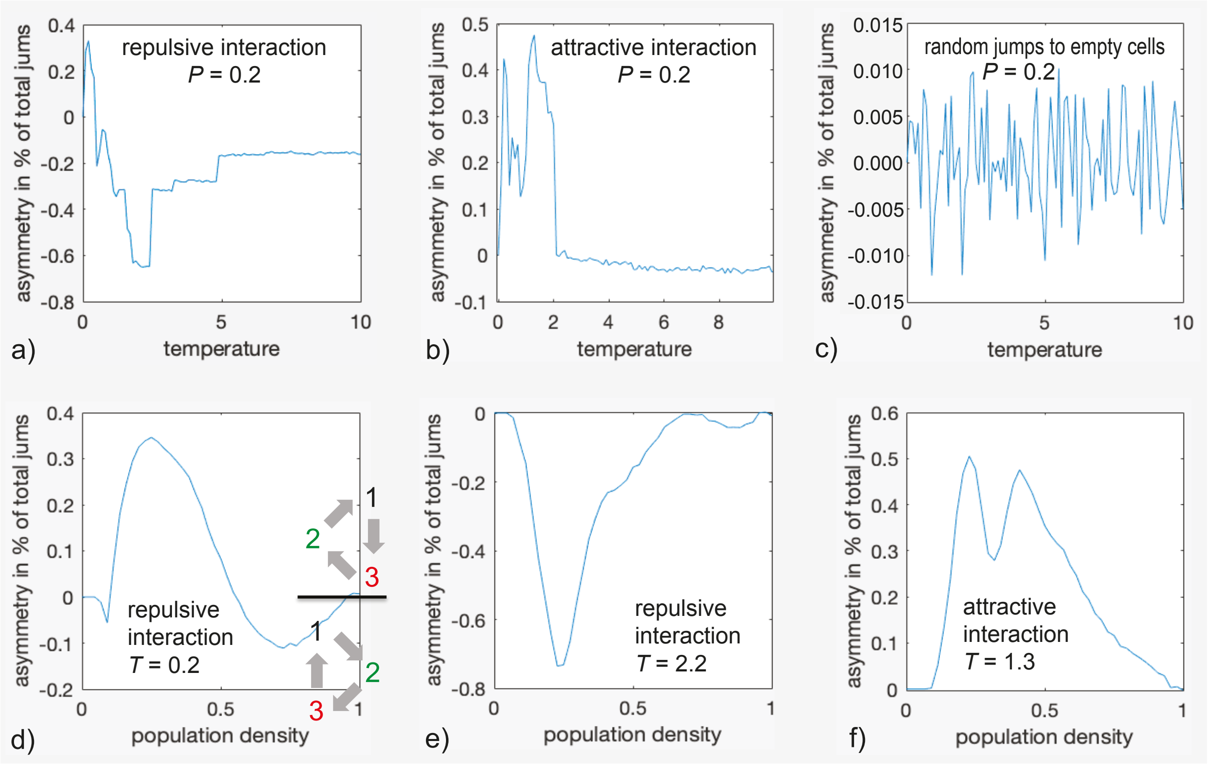

Figure S3. Asymmetry parameters a_{sy} for diffusion in and out of the grain pore depicted in Fig. 3a as a function of relative temperature T (top row) at a population density of 0.2 and relative pressure or population density P (bottom row) at different temperatures. a) $a_{sy}(T)$ for repulsive interaction. b) $a_{sy}(T)$ for attractive interaction. c) $a_{sy}(T)$ for jumps chosen randomly from all free neighbor cells. d) $a_{sy}(P)$ for repulsive interaction at $T = 0.2$. e) $a_{sy}(P)$ for repulsive interaction at $T = 2.2$. f) $a_{sy}(P)$ for attractive interaction at $T = 1.3$.

Population density distributions for different pores and thermodynamic parameters

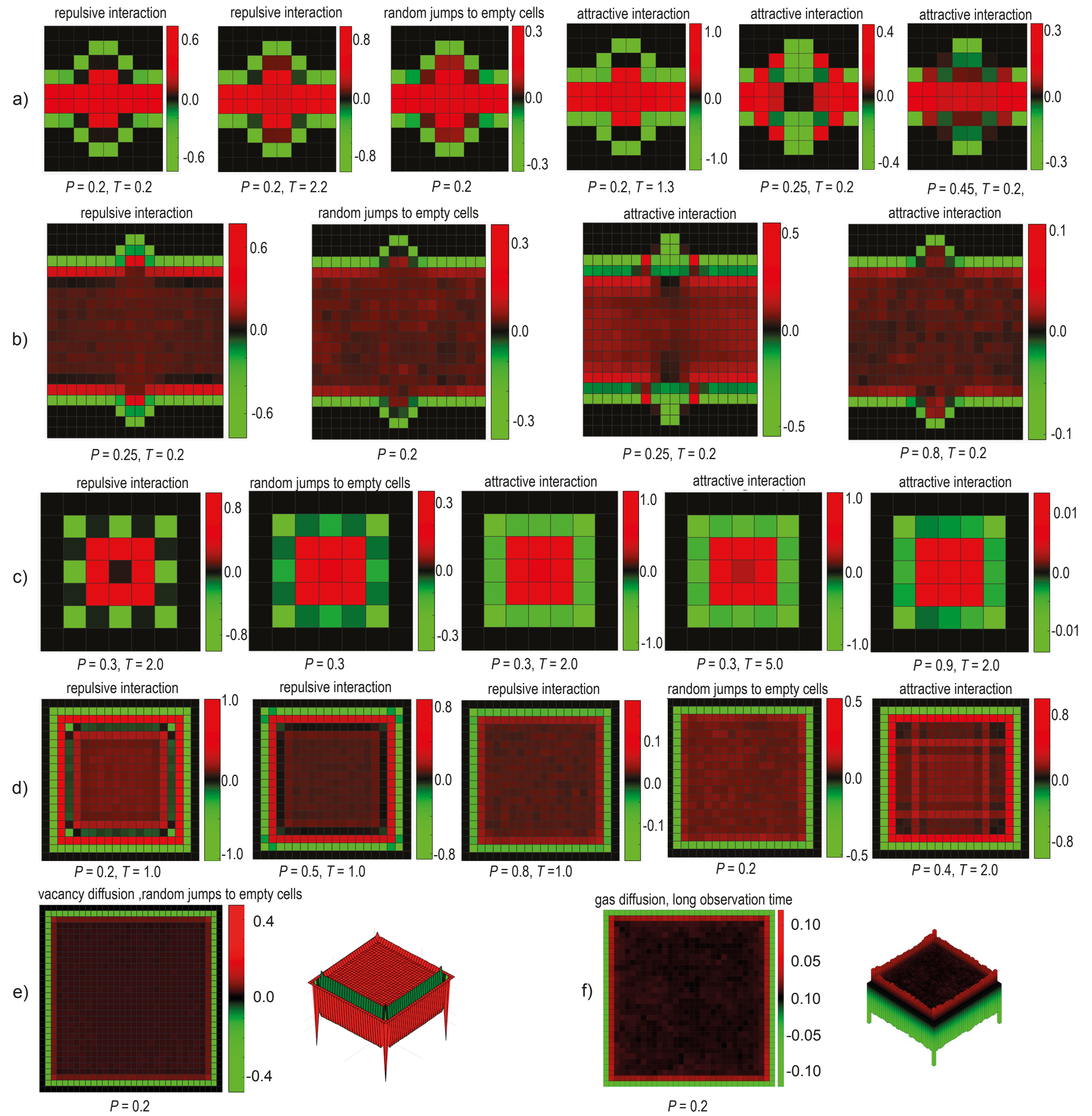

Figure S4. Maps showing the deviations of the particle density from its mean across the pore. a,b) Model for a porous solid, 10^7 jumps. c-e) Square pore, 10^7 jumps. The color scales are different in each plot. The particle concentrations vary more strongly with pressure P than with temperature T. e) Vacancy diffusion in a 32 × 32 pore with random jumps to empty neighbor cells. f) Gas diffusion in a 32 × 32 pore at a long observation time of 1, 10^8 jumps.

Matlab code for vacancy-diffusion simulations

```
% Restricted Diffusion
%
% Define input parameters
ScanP = 0;              % 0 = single pass; 1 = scan Pop; 2 = scan T
pore = 2;               % 1: dent pore (8 x 10); 2: box pore (7 x 7); 3:
parallel planes
Pop = 0.3;              % population-density parameter
T = 0.8;                % temperature parameter
propflag = 0;           % 1: active jump probability, 0: random jumps
Thermo = 0;             % 1: positive DU; 0: DA = 0; else: negative DU
f1 = 1;                 % scale factor for force among particles
f2 = 1;                 % scale factor for force by wall
f3 = 1;                 % scale factor for force by active site
Ny = 7;                 % number of cells in y direction
Nx = 7;                 % number of cells in x direction
DeltaP = 0.04;          % population-density increment
kB = 1;                 % thermal energy constant
DeltaT = 0.04;          % temperature increment
Njump = 10000000;       % number of jumps
% Constants and derived parameters
Nx1 = round(Nx/2);
Ny1 = round(Ny/2);
Nyc = 3;                % detection cell number in y direction
Nxc = 3;                % detection cell number in x direction
Nya = 1;                % active site cell number in y direction
Nxa = 4;                % acive site cell number in x direction
root2 = sqrt(2);
fc = 1/(2*root2);       % scale factor for force from corner cells
nix=zeros(2,1);
% SuperLoop for parameter variation
SLcount = 0;            % number of parameter variations: default is 0
SLn0 = 1;               % parameter-variation counter
if ScanP == 1 SLcount = 25; end     % vary Pop
if ScanP == 2 SLcount = 50; end     % vary T
SLp = zeros(6,SLcount+1);
if ScanP == 1           % population scan
    Pop = 0;
    SLn0 = 2;
end
if ScanP == 2           % temperature scan
    T = 0;
    SLn0 = 2;
end
for SLn = SLn0:SLcount+1% parameter-variation loop starts here *****
    if ScanP == 1
        Pop = Pop + DeltaP;
        SLp(1,SLn) = Pop;
    end
    if ScanP == 2
        T = T + DeltaT;
        SLp(1,SLn) = T;
    end
    k = zeros(3,3);     % kinetic matrix
    tau = 0;            % initiate calculation of autocorrelation function
    taumax = 18*Nx;
    if Ny > Nx taumax = 18*Ny; end
    p2 = 2;             % determine next power of 2 larger than taumax
    while p2 < taumax p2 = p2*2; end
    taumax = p2;
    acorr = zeros(taumax,1);
    noisevec = zeros(taumax,1);
```

```
% Set up pore space with boundaries
    space = zeros(Ny,Nx);
    if pore > 2                     % parallel planes
        space(1,:) = 2;
        space(Ny,:) = 2;
        space(1,Nxa) = 3;           % active site
        space(Ny,Nxa) = 3;          % active site
    end
    if pore == 2                    % box pore
        space(1,:) = 2;
        space(Ny,:) = 2;
        space(:,1) = 2;
        space(:,Nx) = 2;
        space(Nya,Nxa) = 3;             % active site
    end
    if pore < 2                     % dent pore
        space(1,:) = 2;
        space(2,:) = 2;
        space(3,:) = 2;
        space(Ny-2,:) = 2;
        space(Ny-1,:) = 2;
        space(Ny,:) = 2;
        if Ny >= 8                  % define holes
            if Nx >= 6
                space(3,Nx1-1) = 0;
                space(3,Nx1) = 0;
                space(3,Nx1+1) = 0;
                space(3,Nx1+2) = 0;
                space(2,Nx1-1) = 3;
                space(2,Nx1) = 0;
                space(2,Nx1+1) = 0;
                space(2,Nx1+2) = 3;
                space(1,Nx1-1) = 3;
                space(1,Nx1) = 3;
                space(1,Nx1+1) = 3;
                space(1,Nx1+2) = 3;
                space(Ny-2,Nx1-1) = 0;
                space(Ny-2,Nx1) = 0;
                space(Ny-2,Nx1+1) = 0;
                space(Ny-2,Nx1+2) = 0;
                space(Ny-1,Nx1-1) = 3;
                space(Ny-1,Nx1) = 0;
                space(Ny-1,Nx1+1) = 0;
                space(Ny-1,Nx1+2) = 3;
                space(Ny,Nx1-1) = 3;
                space(Ny,Nx1) = 3;
                space(Ny,Nx1+1) = 3;
                space(Ny,Nx1+2) = 3;
            end
        end
    end
    emptypore = space;              % keep empty pore for reference
% Count and populate available cells
    nc0 = 0;                        % count available cells
    for nx = 1:Nx
        for ny = 1:Ny
            if space(ny,nx) == 0 nc0 = nc0 + 1; end
        end
    end
    noc = round(Pop*nc0);           % number of cells to be occupied
    if noc == nc0 noc = nc0-1; end
    if noc == 0 noc = 1; end
    cellvec = zeros(noc,3);         % track occupied cells
```

```
    n = 0;
        while n < noc
            nxi = randi(Nx);
            nyi = randi(Ny);
            if space(nyi,nxi) == 0
                space(nyi,nxi)=1;   % populate
                n = n+1;
                cellvec(n,1) = n;   % track occupied cells
                cellvec(n,2) = nyi;
                cellvec(n,3) = nxi;
            end
        end
    poc = -noc/nc0;
    puc = 1+poc;
    avspace = space;            % initiate summing occupation maps
% Display cell population
    subplot(3,2,1)
    heatmap(space);
    colormap(redgreencmap)
    title('initial population');
    pause(0.1);
    njactive = 0;               % number of active jumps
for njump = 1:Njump             % BEGIN JUMP LOOP
    ncell0 = randi(noc);
    nyi = cellvec(ncell0,2);
    nxi = cellvec(ncell0,3);
% Determine force on cell
    f = [0,0];          % force from all neighbors proportional to 1/(r*r)
    nxip1 = nxi+1; if nxip1 > Nx nxip1 = 1; end
    nxim1 = nxi-1; if nxim1 < 1 nxim1 = Nx; end
    nyip1 = nyi+1; if nyip1 > Ny nyip1 = 1; end
    nyim1 = nyi-1; if nyim1 < 1 nyim1 = Ny; end
nocn = 0;                    % count occupied neighbor cells
ki = 1;                      % identify initial jump environment
c1 = space(nyim1,nxip1);     % possible final position 1
c2 = space(nyi,nxip1);       % possible final position 2
c3 = space(nyip1,nxip1);     % possible final position 3
c4 = space(nyip1,nxi);       % possible final position 4
c5 = space(nyip1,nxim1);     % possible final position 5
c6 = space(nyi,nxim1);       % possible final position 6
c7 = space(nyim1,nxim1);     % possible final position 7
c8 = space(nyim1,nxi);       % possible final position 8
if c1 > 0 nocn=nocn+1;
    if c1 < 2 f = f + [1,-1]*fc*f1; end
    if c1 == 2 f = f + [1,-1]*fc*f2; end
    if c1 > 2 f = f + [1,-1]*fc*f3; end
    if c1 > ki ki = c1; end
end
if c2 > 0 nocn=nocn+1;
    if c2 < 2 f = f + [1,0]*f1; end
    if c2 == 2 f = f + [1,0]*f2; end
    if c2 > 2 f = f + [1,0]*f3; end
    if c2 > ki ki = c2; end
end
if c3 > 0 nocn=nocn+1;
    if c3 < 2 f = f + [1,1]*fc*f1; end
    if c3 == 2 f = f + [1,1]*fc*f2; end
    if c3 > 2 f = f + [1,1]*fc*f3; end
    if c3 > ki ki = c3; end
end
if c4 > 0 nocn=nocn+1;
    if c4 < 2 f = f + [0,1]*f1; end
    if c4 == 2 f = f + [0,1]*f2; end
```

```
    if c4 > 2 f = f + [0,1]*f3; end
    if c4 > ki ki = c4; end
end
if c5 > 0 nocn=nocn+1;
    if c5 < 2 f = f + [-1,1]*fc*f1; end
    if c5 == 2 f = f + [-1,1]*fc*f2; end
    if c5 > 2 f = f + [-1,1]*fc*f3; end
    if c5 > ki ki = c5; end
end
if c6 > 0 nocn=nocn+1;
    if c6 < 2 f = f + [-1,0]*f1; end
    if c6 == 2 f = f + [-1,0]*f2; end
    if c6 > 2 f = f + [-1,0]*f3; end
    if c6 > ki ki = c6; end
end
if c7 > 0 nocn=nocn+1;
    if c7 < 2 f = f + [-1,-1]*fc*f1; end
    if c7 == 2 f = f + [-1,-1]*fc*f2; end
    if c7 > 2 f = f + [-1,-1]*fc*f3; end
    if c7 > ki ki = c7; end
end
if c8 > 0 nonc=nocn+1;
    if c8 < 2 f = f + [0,-1]*f1; end
    if c8 == 2 f = f + [0,-1]*f2; end
    if c8 > 2 f = f + [0,-1]*f3; end
    if c8 > ki ki = c8; end
end
% Determine displacement-energy and -entropy changes
DU = zeros(8,1);
DS = zeros(8,1);
p = zeros(8,2);                 % track cell numbers and jump probabilities
nx = nxi; ny = nyi;             % initial position
Sinitial = myentropy(space,Nx,nx,ny,root2);
space(nyi,nxi) = 0;             % jump from here
if c1 == 0
    DU(1) = f*[1;-1];
    nx = nxip1; ny = nyim1;     % final position 1
    DS(1) = myentropy(space,Nx,nx,ny,root2)- Sinitial;
end
if c2 == 0
    DU(2) = f*[1;0];
    nx = nxip1; ny = nyi;       % final position 2
    DS(2) = myentropy(space,Nx,nx,ny,root2)- Sinitial;
end
if c3 == 0
    DU(3) = f*[1;1];
    nx = nxip1; ny = nyip1;     % final position 3
    DS(3) = myentropy(space,Nx,nx,ny,root2)- Sinitial;
end
if c4 == 0
    DU(4) = f*[0;1];
    nx = nxi; ny = nyip1;       % final position 4
    DS(4) = myentropy(space,Nx,nx,ny,root2)- Sinitial;
end
if c5 == 0
    DU(5) = f*[-1;1];
    nx = nxim1; ny = nyip1;     % final position 5
    DS(5) = myentropy(space,Nx,nx,ny,root2)- Sinitial;
end
if c6 == 0
    DU(6) = f*[-1;0];
    nx = nxim1; ny = nyi;       % final position 6
    DS(6) = myentropy(space,Nx,nx,ny,root2)- Sinitial;
```

```
end
if c7 == 0
    DU(7) = f*[-1;-1];
    nx = nxim1; ny = nyim1;      % final position 7
    DS(7) = myentropy(space,Nx,nx,ny,root2)- Sinitial;
end
if c8 == 0
    DU(8) = f*[0;-1];
    nx = nxi; ny = nyim1;        % final position 8
    DS(8) = myentropy(space,Nx,nx,ny,root2)- Sinitial;
end
space(nyi,nxi) = 1;              % jump from here
% Calculate displacement probabilities
if Thermo ~= 0                   % with thermodynamic contraints
    if Thermo == 1 DA = DU - T*DS;
    else DA = -DU - T*DS; end
else DA = zeros(8,1);
end
p(:,1) = exp(-DA/(kB*T));
p(:,2) = 1:8;
if propflag ==1
    if c1 ~= 0 p(1,1) = 0; end  % exclude occupied destination cells
    if c2 ~= 0 p(2,1) = 0; end
    if c3 ~= 0 p(3,1) = 0; end
    if c4 ~= 0 p(4,1) = 0; end
    if c5 ~= 0 p(5,1) = 0; end
    if c6 ~= 0 p(6,1) = 0; end
    if c7 ~= 0 p(7,1) = 0; end
    if c8 ~= 0 p(8,1) = 0; end
end
% Sort displacement probabilities and determine destination-cell number
P = sortrows(p,'descend');
kf = ki;
n1 = 1;
if P(1,1) >= 1                   % if no energetic constraints for jump
    for nc = 2:8
        if P(nc,1) >= 1 n1 = n1+1; end
    end
else
    for nc = 2:8
        if P(1,1) == P(nc,1) n1 = n1+1; end
    end
end
ncell = P(randi(n1),2);              % randomly pick the destination cell
% Identify destination cell
space(nyi,nxi) = 0;                  % jump from here *******
if ncell > 0
    if ncell == 1 nyi=nyim1; nxi=nxip1; end
    if ncell == 2 nxi=nxip1; end
    if ncell == 3 nyi=nyip1; nxi=nxip1; end
    if ncell == 4 nyi=nyip1; end
    if ncell == 5 nyi=nyip1; nxi=nxim1; end
    if ncell == 6 nxi=nxim1; end
    if ncell == 7 nyi=nyim1; nxi=nxim1; end
    if ncell == 8 nyi=nyim1; end
end
jumpflag = 1;                        % default: jump is possible
if space(nyi,nxi) ~= 0               % jump not possible
    nyi = cellvec(ncell0,2);
    nxi = cellvec(ncell0,3);
    jumpflag = 0;
end
space(nyi,nxi) = 1;                  % jump to there *******
```

```
cellvec(ncell0,2) = nyi;              % update cell vector
cellvec(ncell0,3) = nxi;
% Display cell population
njactive = njactive +1;
if njactive <= 10
    subplot(3,2,1)
    heatmap(space);
    colormap(redgreencmap)
    title(['jump ',num2str(njump)]);
    pause(0.1);
end
% Identify destination-cell environment
nxip1 = nxi+1; if nxip1 > Nx nxip1 = 1; end
nxim1 = nxi-1; if nxim1 < 1 nxim1 = Nx; end
nyip1 = nyi+1; if nyip1 > Ny nyip1 = 1; end
nyim1 = nyi-1; if nyim1 < 1 nyim1 = Ny; end
kf = 1;
c1 = space(nyim1,nxip1); if c1 > kf, kf = c1; end
c2 = space(nyi,nxip1); if c2 > kf, kf = c2; end
c3 = space(nyip1,nxip1); if c3 > kf, kf = c3; end
c4 = space(nyip1,nxi); if c4 > kf, kf = c4; end
c5 = space(nyip1,nxim1); if c5 > kf, kf = c5; end
c6 = space(nyi,nxim1); if c6 > kf, kf = c6; end
c7 = space(nyim1,nxim1); if c7 > kf, kf = c7; end
c8 = space(nyim1,nxi); if c8 > kf, kf = c8; end
% Update kinetix matrix
    if propflag == 0 k(kf,ki) = k(kf,ki) + 1;
    else if jumpflag ~= 0 k(kf,ki) = k(kf,ki) + 1; end
    end
if ScanP == 0              % compute noise autocorrelation
    if space(Nyc,Nxc) > 0
        noise = puc;       % default: position autocorrelation
    else noise = poc;
    end
    avspace = avspace + space;       % determine mean population map
    for ntau = 2:taumax noisevec(ntau-1) = noisevec(ntau); end
    noisevec(taumax) = noise;        % record noise
    for ntau = 1:taumax              % update autocorrelation function
        acorr(ntau)=acorr(ntau)+noisevec(taumax)*noisevec(taumax+1-ntau);
    end
end
end                        %%%%%% END JUMP LOOP
% Show results
pco = 100*noc/nc0;  % percentage of occupied cells
ksum = sum(sum(k)); % total number of jumps
k = k*100/ksum;     % normalize kinetic matrix to % of jumps
disp(num2str(datestr(now,'dd/mm/yy-HH:MM')));   % show date & time
disp(['occupied cells: # ',num2str(noc),', % ',num2str(pco)]);
disp(['# jumps: ',num2str(ksum)]);
disp(['P: ',num2str(Pop),', T: ',num2str(T)]);
asy1 = k(1,2)-k(2,1); asy2 = k(1,3)-k(3,1); asy3 = k(2,3)-k(3,2);
disp(['k12-k21=',num2str(asy1),', k13-31=',num2str(asy2),', k23-
k32=',num2str(asy3)]);
disp('normalized kinetic matrix (number of jumps in %)');
disp(k);
SLp(2,SLn) = asy1;       % wrap up parameter-variation loop
if ScanP == 0
    acorr = acorr/ksum;
    avspace = avspace*nc0/(ksum*noc);
    spacesum = 0;
    for ny = 1:Ny
        for nx = 1:Nx
```

```
            if emptypore(ny,nx) == 0 spacesum = spacesum + avspace(ny,nx);
end
        end
    end
    spacesum = spacesum/nc0;
    maxocc = 0;                     % extrema of avspace
    minocc = 0;
    for ny = 1:Ny                   % set boundary to 1
        for nx = 1:Nx
            if emptypore(ny,nx) > 0, avspace(ny,nx) = 0; end
            if emptypore(ny,nx) == 0 avspace(ny,nx) = avspace(ny,nx) -
spacesum;  end
            if avspace(ny,nx) < minocc minocc = avspace(ny,nx); end
            if avspace(ny,nx) > maxocc maxocc = avspace(ny,nx); end
        end
    end
    absmax = maxocc;
    if -minocc > maxocc absmax = -minocc; end
    subplot(3,2,2)
    heatmap(avspace);
    caxis([-absmax,absmax]);
    colormap(redgreencmap)
    title('variation of average cell population');
    xlabel(['Thermo ',num2str(Thermo),', Pop ',num2str(Pop),', T
',num2str(T),', asy ',num2str(asy3)]);
    subplot(3,2,3)                  % plot autocorrelation function
    ylim([0,0.3]);                  % ****************
    plot(acorr);
    xlabel('time delay');
    ylabel('autocorrelation');
    ntau = 2^nextpow2(taumax);
    as = fft(acorr,ntau);
    ass = complex(zeros(ntau));
    for n = 1:ntau/2                % reshuffle
        ass(n) = as(ntau/2 + n);
        ass(ntau/2 + n) = as(n);
    end
    xaxis = zeros(1,ntau);
    for n=1:ntau xaxis(n) = n-ntau/2-1; end   % xaxis for subplots
    ylim([0,0.3]);                  %****************
    subplot(3,2,4)                  % plot FFT of autocorrelation function
    plot(xaxis,real(ass));          % real part
    xlabel('frequency');
    ylabel('spectral amplitude');
    xlim([-ntau/2-1 ntau/2]);
    subplot(3,2,5)                  % plot FFT of autocorrelation function
    plot(xaxis,imag(ass));          % imaginary part
    xlim([-ntau/2-1 ntau/2]);
    xlabel('frequency');
    ylabel('spectral amplitude');
    xlim([-ntau/2-1 ntau/2]);
    subplot(3,2,6)                  % plot nothing
    avs = zeros(Ny+2,Nx+2);
    for nx=1:Nx
        for ny=1:Ny
            avs(ny+1,nx+1) = avspace(ny,nx);
        end
    end
    surf(avs);
end
end
%%%%%% End parameter-variation loop and show result %%%%%%
if ScanP > 0
```

```
    x = zeros(SLcount+1);
    y = zeros(SLcount+1);
    for SLn = 1:SLcount+1
        x(SLn) = SLp(1,SLn);     % poulation or temperature
        y(SLn) = SLp(2,SLn);     % asymmetry of exchange matrix
    end
    subplot(3,2,2)               % plot nothing
    plot(nix);
    xlabel('nix');
    ylabel('nix');
    title(['Thermo ',num2str(Thermo),' Pop ',num2str(Pop),' T
',num2str(T),' asy ',num2str(asy3)]);
    subplot(3,2,3)
    plot(x,y);
    if ScanP == 1 xlabel('population density'); end
    if ScanP == 2 xlabel('temperature'); end
    ylabel('asymmetry in % of total jums');
end

function S = myentropy(space,Nx,nx,ny,root2)
    S = 0;
    nxp1 = nx+1; if nxp1 > Nx nxp1 = 1; end
    nxm1 = nx-1; if nxm1 < 1 nxm1 = Nx; end
    nyp1 = ny+1;
    nym1 = ny-1;
    if space(nym1,nxp1) == 0 S = S + root2; end
    if space(ny,nxp1) == 0 S = S + 1; end
    if space(nyp1,nxp1) == 0 S = S + root2; end
    if space(nyp1,nx) == 0 S = S + 1; end
    if space(nyp1,nxm1) == 0 S = S + root2; end
    if space(ny,nxm1) == 0 S = S + 1; end
    if space(nym1,nxm1) == 0 S = S + root2; end
    if space(nym1,nx) == 0 S = S + 1; end
end
```

Matlab code for gas-diffusion simulations

```
function [exchangemat,population,asy] =
gasdifsim(speedscale,numparticles,numsteps,dt,diameter,center,numbins)

%%%%% This simulation generates an exchange matrix and population density
%%%%% map for gas particles undergoing elastic collisions. In order the
%%%%% input variables are the scaling factor for the inital velocity
%%%%% profile, the number of particles, the number of time steps, the
%%%%% duration of the time step in arbitrary time units, the diameter of
%%%%% the particles in arbitrary length units, an array of the coordinates
%%%%% for the center of each defect site (i.e. for 7 defects [-0.1 0.1 0.3
%%%%% 0.5 0.7 0.9 1.1], and the number of bins.
```

```
%%%%% Initial condition inputs %%%%

radius(1:numparticles,1) = diameter/2; % same radius for all particles
initialspeed = speedscale.*randn([numparticles 1]); % each particle has a random initial speed

t = 0; % start time
timearray = (t:dt:(t+dt*numsteps))'; % full time array
step = 1 % iteration step
exchangemat(3,3,numbins+2) = 0; % creates a set of 3x3 matrices, one for each defect site

bincenter = (1/(2*numbins)):(1/numbins):1;


%%%%% Generates randomized particles %%%%%

%Randomizing initial positions and ensuring no overlap of particles

initialpos = round(rand(numparticles,1)*(numbins^2-1))+1;
for ii = 2:numparticles
    while any(initialpos(ii)==initialpos(1:(ii-1)))
        initialpos(ii) = round(rand*(numbins^2-1))+1;
    end
end

initialpos = initialpos/numbins;
binx = ceil(initialpos);
biny = round((initialpos-(binx-1))./diameter);

for ii = 1:numparticles
    initialx(ii,1) = bincenter(binx(ii));
    initialy(ii,1) = bincenter(biny(ii));
end
```

```
    positionx = initialx; positiony = initialy; % Tracking current positions

    theta = 2.*pi.*randn([numparticles 1]); % randomized initial angle of travel
    velocityx = cos(theta).*initialspeed; velocityy = sin(theta).*initialspeed; % x and y velocity components assigned based on random angle calculation

    particles = [positionx,positiony,velocityx,velocityy]; % each row defines a particle with initial positions, velocities, and radius
    pos2d = [positionx,positiony]; % Tracking both current position components in one array


    population = zeros(numbins); % empty matrix to track particle locations at each step
    binlimits = [-0.05 (bincenter(2)-bincenter(1)):(bincenter(2)-bincenter(1)):(1-(bincenter(2)-bincenter(1))) 1.05];
    % Boundary definitions for each grid element, total length of x and y
    % walls goes from 0 to 1 in arbitrary length units. Each grid element is
    % the length of one particle diameter or 1/numbins


    % Populates the population density count with the initial positions.
    % Takes the inital x and y coordinate of each particle and determines
    % which grid element it falls into and updates cooresponding matrix
    % element by +1
    for pp = 1:numparticles
        for ff = 1:(numbins)
            for gg = 1:(numbins)
                if pos2d(pp,1)>binlimits(ff) && pos2d(pp,1)<=binlimits(ff+1) && pos2d(pp,2)>binlimits(gg) && pos2d(pp,2)<=binlimits(gg+1)
                    population(ff,gg) = population(ff,gg) + 1;
                end
            end
```

```
        end
    end


    %%%%% Moving the particles %%%%%

    for hh = 1:numsteps %looping through each step

        step = step+1 %increment to the next time step
        t = t+dt; %current time
        collisionpair = [];


        % Compares the positions of each possible pair of particles. When
        % the coordinates are within 1 particle diameter of each other they
        % are considered to have collided. A list of all colliding pairs is
        % generated.
        for gg = 1:(numparticles-1)
            collidingparticle1 = particles(gg,1:2);
            for jj = (gg+1):numparticles
                collidingparticle2 = particles(jj,1:2);
                if norm(collidingparticle2-collidingparticle1) < diameter
                    collisionpair = [collisionpair;gg jj];
                end
            end
        end



        for ll = 1:size(collisionpair,1)

            collidingposition1 = particles(collisionpair(ll,1),1:2); % current position of
colliding particle 1
```

```
            collidingposition2 = particles(collisionpair(ll,2),1:2); % current position of
colliding particle 2
            collidingvelocity1 = particles(collisionpair(ll,1),3:4); % current velocity of
colliding particle 1
            collidingvelocity2 = particles(collisionpair(ll,2),3:4); % current velocity of
colliding particle 2


            % Calculation of new velocities for the elastic collision of particles
            dr = collidingposition2 - collidingposition1;
            dv = collidingvelocity2 - collidingvelocity1;
            dvdotdr = dot(dv,dr);
            d = norm(dr)^2;

            newvelocity1 = collidingvelocity1 - dvdotdr./d.*(collidingposition1-
collidingposition2);
            newvelocity2 = collidingvelocity2 - dvdotdr./d.*(collidingposition2-
collidingposition1);


            % Replacing the pre-collision velocities with the
            % post-collision velocities
            velocityx(collisionpair(ll,1)) = newvelocity1(1); velocityx(collisionpair(ll,2)) =
newvelocity2(1);
            velocityy(collisionpair(ll,1)) = newvelocity1(2); velocityy(collisionpair(ll,2)) =
newvelocity2(2);
            particles = [positionx,positiony,velocityx,velocityy];
        end

        for kk = 1:numparticles
            %checking x boundaries, deflect if collision
            if ((particles(kk,1) - 0) < radius(1) && velocityx(kk) < 0) || ((1 - particles(kk,1))
< radius(1) && velocityx(kk) > 0)
                velocityx(kk) = -1.*velocityx(kk);
```

```
            %checking y boundaries, deflect if collision
            elseif ((particles(kk,2) - 0) < radius(1) && velocityy(kk) < 0) || ((1 - particles(kk,2)) < radius(1) && velocityy(kk) > 0)
                velocityy(kk) = -1.*velocityy(kk);
            end
        end


        % Move particles to their next position and record
        positionx = positionx + velocityx.*dt; positiony = positiony + velocityy.*dt;
        particles = [positionx,positiony,velocityx,velocityy];
        pos2d((numparticles+1):(2*numparticles),:) = [positionx positiony];




        for jj = 1:numparticles

            % Update population density matrix with the new positions (see
            % line 46)
            for ff = 1:numbins
                for gg = 1:numbins
                    if pos2d(jj+numparticles,1)>binlimits(ff) && pos2d(jj+numparticles,1)<=binlimits(ff+1) && pos2d(jj+numparticles,2)>binlimits(gg) && pos2d(jj+numparticles,2)<=binlimits(gg+1)
                        population(ff,gg) = population(ff,gg) + 1;
                    end
                end
            end



    %%%%% Counting for the Exhange matrix %%%%%

```

```
    % Site 1 is in the body of the pore, Site 2 is the wall, and Site 3
    % is the defect site.

      for defectsite = 1:(numbins+2)

        % Particle is found at a Site 3 grid space at previous time step
        if (pos2d(jj,1)<diameter && (pos2d(jj,2)<(center(defectsite)+3*diameter/2) &&
pos2d(jj,2)>(center(defectsite)-3*diameter/2)))

          % Particle is still in a Site 3 grid space on current step
          if (pos2d(numparticles+jj,1)<diameter &&
(pos2d(numparticles+jj,2)<(center(defectsite)+3*diameter/2) &&
pos2d(numparticles+jj,2)>(center(defectsite)-3*diameter/2)))
            exchangemat(3,3,defectsite) = exchangemat(3,3,defectsite) + 1;

          % Particle moved to a Site 1 grid space on current step
          elseif (pos2d(numparticles+jj,1)>=diameter && (1-
pos2d(numparticles+jj,1))>=diameter) && (pos2d(numparticles+jj,2)>=diameter &&
(1-pos2d(numparticles+jj,2))>=diameter)
            exchangemat(3,1,defectsite) = exchangemat(3,1,defectsite) + 1;

          % Particle moved to a Site 2 grid space on current step
          else
            exchangemat(3,2,defectsite) = exchangemat(3,2,defectsite) + 1;
          end


        % Particle is found at a Site 1 grid space at previous time step
        elseif (pos2d(jj,1)>=diameter && (1-pos2d(jj,1))>=diameter) &&
(pos2d(jj,2)>=diameter && (1-pos2d(jj,2))>=diameter)

          % Particle is still in a Site 1 grid space on current step
```

```
            if (pos2d(numparticles+jj,1)>=diameter && (1-
pos2d(numparticles+jj,1))>=diameter) && (pos2d(numparticles+jj,2)>=diameter &&
(1-pos2d(numparticles+jj,2))>=diameter)
               exchangemat(1,1,defectsite) = exchangemat(1,1,defectsite) + 1;

            % Particle moved to a Site 3 grid space on current step
            elseif (pos2d(numparticles+jj,1)<diameter &&
(pos2d(numparticles+jj,2)<(center(defectsite)+3*diameter/2) &&
pos2d(numparticles+jj,2)>(center(defectsite)-3*diameter/2)))
               exchangemat(1,3,defectsite) = exchangemat(1,3,defectsite) + 1;

            % Particle moved to a Site 2 grid space on current step
            else
               exchangemat(1,2,defectsite) = exchangemat(1,2,defectsite) + 1;
            end


         % Particle is found at a Site 2 grid space at previous time step
         else

            % Particle moved to a Site 1 grid space on current step
            if (pos2d(numparticles+jj,1)>=diameter && (1-
pos2d(numparticles+jj,1))>=diameter) && (pos2d(numparticles+jj,2)>=diameter &&
(1-pos2d(numparticles+jj,2))>=diameter)
               exchangemat(2,1,defectsite) = exchangemat(2,1,defectsite) + 1;

            % Particle moved to a Site 3 grid space on current step
            elseif (pos2d(numparticles+jj,1)<diameter &&
(pos2d(numparticles+jj,2)<(center(defectsite)+3*diameter/2) &&
pos2d(numparticles+jj,2)>(center(defectsite)-3*diameter/2)))
               exchangemat(2,3,defectsite) = exchangemat(2,3,defectsite) + 1;

            % Particle is still in a Site 2 grid space on current step
            else
```

```
                exchangemat(2,2,defectsite) = exchangemat(2,2,defectsite) + 1;
            end
        end
    end

    end

    % Current time step is moved to previous time step for next iteration
    pos2d(1:numparticles,:) = pos2d((numparticles+1):(2*numparticles),:);

end


% Heat map for the population density, normalized by the average
% population and centered about 0
figure
heatmap(population./mean(population,'all')-1)
colorbar

for ii = 1:7
    asy(ii,1) = (exchangemat(1,2,ii)-
exchangemat(2,1,ii)).*100./sum(exchangemat(:,:,ii),'all');
    asy(ii,2) = (exchangemat(2,3,ii)-
exchangemat(3,2,ii)).*100./sum(exchangemat(:,:,ii),'all');
    asy(ii,3) = (exchangemat(1,3,ii)-
exchangemat(3,1,ii)).*100./sum(exchangemat(:,:,ii),'all');
end

end
```
